# AKT kinases as therapeutic targets

**DOI:** 10.1186/s13046-024-03207-4

**Published:** 2024-11-29

**Authors:** Dalal Hassan, Craig W. Menges, Joseph R. Testa, Alfonso Bellacosa

**Affiliations:** 1 Nuclear Dynamics and Cancer Program, Cancer Epigenetics Institute, Institute for Cancer Research, Fox Chase Cancer Center, 333 Cottman Avenue, Philadelphia, PA 19111 USA; 2 Cancer Prevention and Control Program, Fox Chase Cancer Center, 333 Cottman Avenue, Philadelphia, PA 19111 USA; 3 Thomas Jefferson University, 901 Walnut St, Philadelphia, PA 19107 USA

**Keywords:** AKT kinases, Cancer, Therapy, Overgrowth syndromes, Inflammation

## Abstract

AKT, or protein kinase B, is a central node of the PI3K signaling pathway that is pivotal for a range of normal cellular physiologies that also underlie several pathological conditions, including inflammatory and autoimmune diseases, overgrowth syndromes, and neoplastic transformation. These pathologies, notably cancer, arise if either the activity of AKT or its positive or negative upstream or downstream regulators or effectors goes unchecked, superimposed on by its intersection with a slew of other pathways. Targeting the PI3K/AKT pathway is, therefore, a prudent countermeasure. AKT inhibitors have been tested in many clinical trials, primarily in combination with other drugs. While some have recently garnered attention for their favorable profile, concern over resistance and off-target effects have continued to hinder their widespread adoption in the clinic, mandating a discussion on alternative modes of targeting. In this review, we discuss isoform-centric targeting that may be more effective and less toxic than traditional pan-AKT inhibitors and its significance for disease prevention and treatment, including immunotherapy. We also touch on the emerging mutant- or allele-selective covalent allosteric AKT inhibitors (CAAIs), as well as indirect, novel AKT-targeting approaches, and end with a briefing on the ongoing quest for more reliable biomarkers predicting sensitivity and response to AKT inhibitors, and their current state of affairs.

## Introduction

The discovery of *Akt* as an oncogene dates to about four decades ago, when a novel transforming retrovirus, isolated from an AKR mouse T cell lymphoma [1], was found to carry transduced sequences of cellular origin [1]. Our collaborative work with Philip Tsichlis and Stephen Staal led to cloning the viral oncogene v-*akt* as the oncogene transduced by the AKT8 retrovirus (originally from an AKR mouse T cell lymphoma) [2]. The oncoprotein encoded by this retrovirus was named v-Akt. It consisted of viral gag sequences fused to a kinase related to protein kinase C, containing a Src homology 2 (SH2)-like domain (c-Akt, of cellular origin). Using different strategies to discover novel protein kinases, two other research groups independently cloned the identical cellular sequence at about the same time [3, 4]. The oncogenic potential of v-Akt arose from the myristylation of the gag protein at the N-terminus, resulting in constitutive activation of v-Akt [5]. AKT is now known to consist of three highly conserved cellular homologs defined in humans as AKT1, AKT2, and AKT3 (reviewed in [6]).

Since then, many attempts have been made to target AKT to treat cancer, as it plays a pivotal role in many defining features of malignant cells [7–10]. Despite the significant amount of progress made with the development of AKT inhibitors, the therapeutic benefit gleaned from these drugs, mainly in the form of dual therapies, is variable. Toxicity resulting from their non-selectivity due to AKT, ubiquitous expression [11] and structural homology with other functionally important proteins [2–4, 12], and the development of resistance because of crosstalk between AKT and a plethora of other pathways, are ongoing issues. There is a dire need to identify biomarkers of sensitivity, response, and resistance that should be individualized for each tumor and patient to optimize the therapeutic window of these drugs, which requires a deeper understanding of AKT’s perplexing biology. Clarifying the roles that different AKT isoforms play in cancer-specific tumor initiation and progression remains an unmet need. As such, indirectly targeting AKT by re-directing our attention to the metabolic, oxidative, and proteotoxic consequences of AKT hyperactivation, even autophagy, maybe a better, albeit less practical approach, since non-tumoral cells rely heavily on these built-in mechanisms for their normal functioning.

Here, we begin with an overview of AKT and isoform structure, regulation, and function and briefly discuss the mechanisms by which AKT’s function can be perturbed. We then describe the roles of the three AKT isoforms in overgrowth syndromes and cancer and their effects on the immune system, particularly on T cells and macrophages, and discuss the implications of targeting specific isoforms for cancer and inflammatory/autoimmune disease treatment and prevention. We then touch upon biomarkers of sensitivity and response to AKT inhibitors, AKT inhibitors currently being tested in clinical trials, with a focus on capivasertib, problems with their usage, how to maximize efficacy while avoiding their many toxicities and end with a discussion on novel therapeutic angles from which AKT can be targeted.

## AKT structure, function & regulation

Akt, also known as protein kinase B (PKB), is a 57-kDa integral kinase and signaling node that belongs to the protein kinase A, kinase G, and kinase C (AGC) superfamily of serine/threonine kinases, which includes the ribosomal S6 protein kinase and serum-glucocorticoid regulated kinases (SGK) [13] Under conditions of homeostasis, Akt responds to extracellular cues by positively regulating cell survival, growth, metabolism, and cytoplasmic reorganization and migration, via the phosphorylation of serine and threonine residues of many downstream substrates [14, 15]. The three human AKT genes, *AKT1*, *AKT2*, and *AKT3*, found on chromosomes 14q32 [16], 19q13 [17], and 1q44 [18] in the mammalian genome, share a canonical structure consisting of an N-terminal pleckstrin homology (PH) domain that autoinhibits AKT in the basal state by interacting intramolecularly with the kinase domain, an α-helical linker domain, a central catalytic (kinase) domain, which contains a regulatory threonine residue in its activation loop, and a C-terminal hydrophobic, proline-rich motif containing a regulatory serine residue. Considerable sequence homology exists among the domains of the three AKT isoforms, but the linker domain is highly divergent [19–24].

Despite possessing a similar structure, each isoform shows varying levels of expression at the mRNA and protein levels in different cells and distinct subcellular localizations [25], implying that they may have different substrate specificities and hence non-overlapping functions, in addition to redundant roles [26]. Their different substrate specificities may also be due, in part, to them having non-redundant, non-canonical motifs, or recognizing substrates with a specific conformation [27]. Alternatively, they may have overlapping motifs, but their different substrate specificities could be the result of post-translational modifications (e.g., phosphorylation) by other kinases, regulation by miRNAs, or extracellular activation (reviewed in [28]). Palladin, for example, has recently been identified as a substrate of AKT1, although its expression is regulated by AKT2 [29]; the phosphorylation of AKT1 on Ser131 in the linker region by casein kinase 2 helps direct AKT1’s specificity for palladin [30].

During embryonic development, all tissues express Akt1, the principal isoform, to a similar degree, whereas Akt2 is predominantly expressed in insulin-sensitive tissues, such as skeletal muscle, liver, and adipose tissue [31], and Akt3 is mainly present in neuronal tissue and testis, and to a lesser extent, in lungs, mammary glands, and adipose tissue [32]. Assigning phenotypic roles to the three isoforms was aided by mouse knockout studies, where it was observed that some *Akt1*^*−/−*^ mice were non-viable, while others showed severe growth retardation and developmental deficits, *Akt2*^*−/−*^ mice developed insulin-sensitive diabetes mellitus, and *Akt3*^*−/−*^ mice had reduced brain sizes and impaired brain development [33, 34]. Other examples showing that the non-overlapping function of the different isoforms is at least partly attributed to their subcellular compartmentalization include findings that isoform-specific knockdown of *AKT* in MDA-MB-231 cells, a human breast cancer cell line, did not force the other isoforms to a different subcellular location [25]. That is not to say that one AKT isoform cannot reside in more than one subcellular compartment, as AKT1 and AKT2 have been detected in the nucleus of breast cancer cells [29], as well as the cytoplasm or mitochondria [26], which makes ascribing a single function to a particular isoform in such cellular contexts quite difficult. In mouse adipocytes, insulin-induced activation of the Glut4 glucose transporter is mainly due to the presence of Akt2 at the plasma membrane. The expression of the E17K variant of Akt1 resulted in the constitutive plasma membrane translocation of Akt1 and the activation of Glut4, abolishing the need for Akt2 [35]. However, the question of why substituting AKT2’s PH domain for AKT1 did not facilitate AKT1’s movement to the plasma membrane [25] or induce cell proliferation and G1/S (cell cycle) progression [36] remains unresolved. It is possible that there are signaling proteins that only recognize isoform-specific PH domains and that these proteins are nestled within specific subcellular compartments [27]. Lending credence to this premise is the fact that T-cell leukemia-1b (TCL1b) is dependent on AKT3’s PH domain for binding to AKT3; transferring AKT1’s PH domain to AKT3 prevented TCL1b from binding to AKT3 [37]. In untransformed fibroblasts, AKT1 promotes migration, and AKT2 has anti-migratory effects, whereas in breast cancer cell lines, the opposite holds true [38]. Therefore, both cell-type and cancer-specific contexts must be accounted for when assigning different roles to Akt isoforms.

Studies supporting overlapping roles for the different Akt isoforms (reviewed in [39]) include those conducted by Chen et al., who showed that haploinsufficiency of *Akt1* in *Akt2*^*−/−*^ mice causes hyperinsulinemia and hyperglycemia and that this is partly due to lipodystrophy and leptin deficiency; hyperinsulinemia and hyperglycemia were reversed in *Akt2*^*−/−*^ and *Akt2*^*−/−*^*;Akt1*^±^ mice when Akt1 was hyperactivated [40]. These results can be extended to humans, where families with inherited, dominant-negative mutations in *AKT2* often develop type II diabetes in combination with lipodystrophy [41, 42].

Akt1, Akt2, and Akt3 appear to be controlled similarly. However, the regulatory serine/threonine residues that undergo inducible phosphorylation differ between the three isoforms (T308/T309/T305 and S473/S474/S472 on Akt1, Akt2, and Akt3, respectively) [43]. All isoforms are basally phosphorylated at Ser124 and Thr450 [19, 44] with inducible phosphorylation taking place when tyrosine kinase, cytokine, B and T-cell, integrin, G-protein-coupled, or toll-like receptors are stimulated in various cell types, for example, consequential to extracellular matrix attachment or stimulation by mitogens [45, 46].

Receptor signaling triggers the activation of the phospholipid phosphatidylinositol-3-phosphate kinase (PI3K), which converts phosphatidylinositol-4,5-bisphosphate (PIP2) to the lipid second messenger, phosphatidylinositol-3,4,5-triphosphate (PIP3) [47]. The binding of PIP3 to the PH domain of Akt is an essential step in Akt activation in that it not only recruits and anchors Akt to the plasma membrane [19, 48], but also promotes the formation of Akt homomultimers [48, 49]. Before PIP3 can bind to Akt, however, Akt is ubiquitinated by tumor necrosis factor receptor associated factor 6 (TRAF6), an E3 ligase, on K8 and K14, within the PH domain, for it to interact with critical adapters, such as JNK-interacting protein 1 (JIP1) and T cell leukemia-1 (TCL1), which facilitate Akt’s recruitment to the plasma membrane [50–52]. Other studies maintain that this ubiquitination occurs on K63 with the help of tumor necrosis factor receptor-associated factor 4 (TRAF4), S-phase kinase associated protein 2 (SKP2), or TRAF6 [50, 51, 53] and that this is promoted by SET domain bifurcated histone lysine methyltransferase 1 (SETDB-1), which methylates Akt1 at lysine 64, paving the way for lysine demethylase 4A to recruit TRAF6 or SKP2 to Akt [54]. An increase in the deubiquitinating enzyme CYLD lysine 63 deubiquitinase (CYLD) and ubiquitin-specific peptidase 1 activity results in Akt deubiquitination and hinders its plasma membrane recruitment [55, 56]. The concurrent binding of PIP3 to the PH domains of Akt and 3-phosphoinositide-dependent protein kinase 1 (PDK-1; gene name: *PDPK1*) at the plasma membrane induces a conformational change in Akt that exposes the activation loop and allows Akt Thr308/T309/T305 to be phosphorylated by PDK-1, partially activating Akt1’s catalytic domain. Mutations occurring in the PH domain may render Akt more likely to bind to PIP3, with subsequent phosphorylation and activation by PDK-1, or less likely to bind to PIP3 [11].

**Fig. 1:**
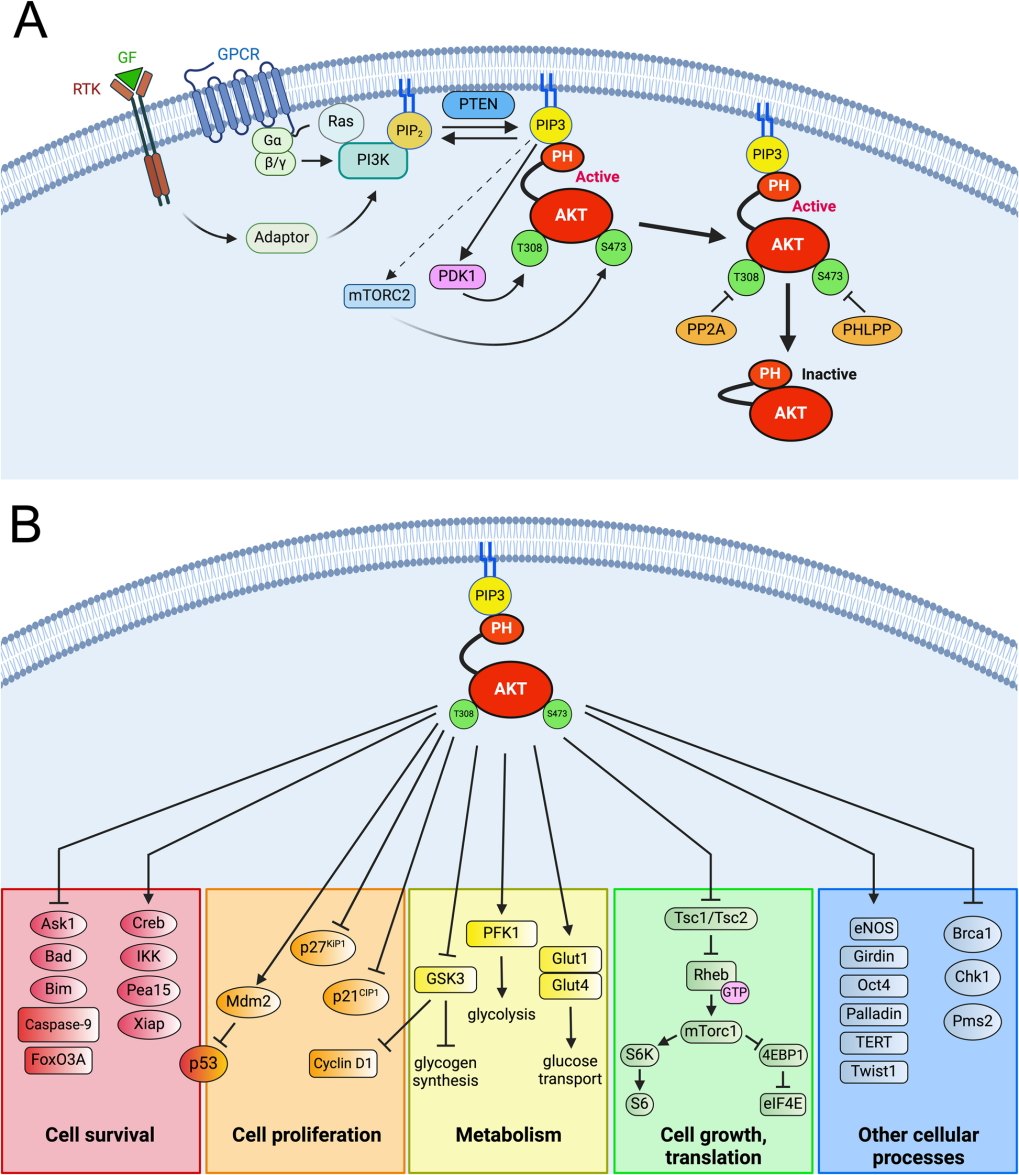
**A** Schematic of AKT activation and inactivation; activating and inactivating steps are indicated by arrows and blunt-ended lines, respectively (modified from [130]). **B** AKT substrates; functional and biological consequences of their phosphorylation. Schematic showing the cellular functions of known AKT substrates. Direct phosphorylation by AKT is indicated by continuous lines, leading to activation (arrow end) or inhibition (blunt end). See main text for details

To become fully activated, Akt must also be phosphorylated on Ser473/S474/S472, usually by the mammalian target of rapamycin complex 2 (mTORC2), whose members include the PDK-2 [57]; mTORC2 can also indirectly activate Akt through a feed-forward mechanism by phosphorylating and activating the insulin receptor (InsR)/insulin-like growth factor receptor (IGF1R) [58, 59] In some cases, however, Akt is auto-phosphorylated on Ser473 [60], or is phosphorylated by PI3K-related kinases, such as protein kinase C-beta II [61], PDK-1, upon PDK-1’s interaction with protein kinase c-related kinase 2 (PRK-2) [62], DNA-dependent protein kinase (DNA-PK) [63, 64] and ataxia telangiectasia mutated (ATM), in response to DNA damage and DNA replication stress in the nucleus [65], or integrin-linked kinase [66]. Because of DNA-damaging agents, the direct activation of Akt by DNA-PK is responsible for chemoradiation treatment resistance. It has recently been shown that DNA-PK can also phosphorylate the mTORC2 subunit, Sin1, allowing Sin1 to interact with the guanine nucleotide exchange factor (GEF), ECT2 [67]. The basic arginine patch in the linker domain can promote Akt1 activation by interacting with phosphorylated S473 at the C-terminus [68]. Although commonly phosphorylated by PDK1 and mTORC2, the regulatory serine/threonine residues in the three Akt isoforms can be directly phosphorylated by IκB kinase epsilon (IKKE) and TANK-binding kinase 1 (TBK1) in a PI3K-dependent, PDK-1-, and mTORC2-independent manner [69–71] The carboxyl-terminal modulatory protein (CTMP), which was once thought to decrease T308 and S473 phosphorylation and Akt activation by binding to Akt’s C-terminal domain [72], has now been shown to be responsible for Akt phosphorylation and activation and is overexpressed in head and neck and breast cancer [73, 74]. A schematic of AKT activation and inactivation is shown in Fig. 1A.

Noteworthy is the fact that the expression of AKT isoforms fused to an N-terminal Src myristylation signal (MGAG residues), which allows Akt to associate with the plasma membrane via a mechanism that precludes the PH domain, is enough to transform embryonic fibroblasts and increase the development of mammary carcinoma when expressed in a transgenic mouse model via the mammary-specific mouse mammary tumor virus (MMTV) promoter [75–77]. The viral oncogene v-*akt* also contained an N-terminally myristoylated GAG fusion [5, 26], further reinforcing this concept. This underscores the importance of both phosphorylation and membrane association as requirements for Akt activation.

Ser477, Thr479, Ser124, and Thr450 are novel phosphorylation sites that can also activate Akt1; the phosphorylation of the former two residues is mediated by mTORC2, or cyclin-dependent kinase 2 (cdk2)/cyclin complex [11, 78]. In addition to activation by phosphorylation, the binding proteins actin, extracellular signal-regulated protein kinase (Erk) 1/2, heat shock proteins (Hsp) 90 and 27, and Posh can activate Akt indirectly by controlling its stability [79].

Once activated, Akt dissociates from the cell membrane and is transported to the cytosol, nucleus, or mitochondria, where it phosphorylates and activates or inhibits numerous downstream effectors, many of which contain the consensus phosphorylation sequence RxRxxS/T, culminating in cell growth, metabolism, migration, survival, cell cycle progression, and angiogenesis, processes that constitute cancer hallmarks and are frequently deregulated in cancer cells [9, 80]. The phosphorylation and inactivation of tuberous sclerosis 1 and 2 (TSC1/2) by Akt leads to the disinhibition of the Ras homolog enriched in the brain (Rheb) and the accumulation of its GTP-bound form, which favors the conversion of mTORC2 to mTORC1. mTORC1 then phosphorylates ribosomal protein p70S6 kinase and eukaryotic translation initiation factor 4E (eIF4E) binding protein-1 (4E-BP1), enabling protein synthesis [34, 81].

Other notable downstream substrates whose activation states are modified by AKT include IκB kinase (IKK) [82], mouse double minute 2 homolog (Mdm2) [83, 84], which promotes the ubiquitination and degradation of p53, the pro-apoptotic proteins BCL-2 associated agonist of cell death (Bad) and caspase-9, the cell cycle inhibitors p27 and p21, glycogen synthase kinase-3 (GSK3) [85] and the forkhead family of transcription factors (FOXO) 1–4 [86], that are retained in the cytoplasm by 14–3-3 proteins when phosphorylated [87]. More recently, the substrate repertoire of AKT has been expanded to include telomeric repeat binding factor 1 (TRF1), a member of the telomere-bound shelterin complex, which is hyperactivated in cancer cells, and endows them with limitless replicative potential [88]. Known AKT substrates and the functional and biological effects of their phosphorylation are listed in Table 1 and illustrated in Fig. 1B. Aside from the plasma membrane, AKT can undergo activation in other subcellular compartments, including the endosome, lysosome, endoplasmic reticulum, and nucleus [89].

**Table 1:** AKT substrates

**Substrate**	**Function**	**Phosphorylation Effect**	**Biological Effect of Phosphorylation**	**Ref.**
**FOXO4**	Transcription factor that induces expression of CDK inhibitor p27, and pro-apoptosis genes	Cytoplasmic retention and/or degradation		[90, 91]
**GSK3α, GSK-3β**	Degrades β-catenin, cyclin D1 and Myc Inhibits glycogen synthesis Regulates apoptosis by destabilizing MCL-1	Inhibition		[92–94]
**p21**^**CIP1**^, **p27**^**KIP1**^	Members of CIP-KIP family of cyclin-dependent kinase (CDK) inhibitors	Cytoplasmic retention	Cell cycle progression, and cell proliferation (including Mdm2, except for c-Raf)	[95, 96]
**USP43**	Represses EGFR in combination with NuRD complex	Cytoplasmic retention		[97]
**USF-1**	Induces the transcription of the oncogene WBP2	Activation		[98]
**c-Raf**	MAP kinase, part of the ERK1/2 pathway	Inhibition		[99]
**Bad**	Pro-apoptotic	Inhibition		[100]
**Bim**	Pro-apoptotic	Inhibition (inactivation or 14-3-3 binding)		[101]
**Procaspase-9**	Pro-apoptotic	Inhibition		[102]
**MST2**	Pro-apoptotic kinase	Inhibition		[103]
**CREB**	Regulates the transcription of anti-apoptosis genes, including bcl-2 and mcl-1	Activation	Survival (including HK-2 & FOXO4)	[104]
**IKKα**	Phosphorylation and ubiquitination of IkB, an inhibitor of NF-κB	Activation		[105, 106]
**FOXO1, FOXO3**	Transcription factors involved in the expression of pro-apoptotic genes, and cell differentiation and metabolism (n/iTreg differentiation)	Cytoplasmic retention and/or degradation		[90, 91]
**YAP1**	Pro-apoptotic	Cytoplasmic retention		[107]
**Mdm2**	Promotes ubiquitination and degradation of p53 when activated	Nuclear translocation		[84]
**ASK-1**	Induces apoptosis via JNK pathway	Inhibition		[108]
**AR**	Nuclear receptor; mediates growth & survival	Activation		[109]
**Palladin**	Actin-bundling protein and scaffold. Inhibits breast cancer cell migration (Akt1)	Activation (Akt1)		[29]
**Twist1**	Upregulates expression of transforming growth factor-β2	Activation		[110]
**Vimentin**	Increases cell migration and invasion	Activation	Migration, invasion & metastasis	[111]
**Girdin**	Promotes lamellipodia formation and cell motility; Increases VEGF-induced angiogenesis	Activation		[112]
**TSC2**	TSC1/TSC2 complex inhibit mTORC1 activity	Inhibition		[113]
**4E-BP1**	Negative regulator of translation	Inhibition	Protein synthesis and cell growth	[114]
**PRAS40**	Negative regulator of mTORC1	Inhibition		[115]
**BRCA1**	DNA repair	Inhibition	Genomic instability	[116]
**TRF-1**	Chromosome-end protection and genomic stability	Activation	Telomere maintenance	[88]
**TBC1D4**	Rab GTPase-activating protein; regulates membrane translocation of GLUT-4	Inhibition (inhibits its GTPase-activating activity)		[117]
**PIKFYVE**	Facilitates membrane translocation of GLUT-4 vesicles	Activation		[118, 119]
**TXNIP**	Negative regulator of GLUT1 and GLUT4 by promoting their endocytosis	Inhibition	Increased glucose uptake and glycolysis (including FOXO1, FOXO3 and GSK-3β)	[120]
**TBC1D1**	Negative regulator of GLUT1 protein expression	Inhibition		[121]
**PFKFB2**	Glycolytic enzyme	Activation		[122]
**HK2**	Glycolytic enzyme, mitochondrial binder and protector, promotes autophagy by inhibiting mTORC1	Activation		[123, 124]
**ACOT4**	Releases free fatty acids from acetyl-CoA	Activation		[125]
**ACLY**	Production of acetyl-CoA from citrate	Activation	Lipid synthesis	[126]
**PDE3B**	Inhibition of lipolysis	Activation		[127]
**eNOS**	Stimulates vasodilation	Activation	Angiogenesis (including Girdin)	[128, 129]

*Legend*: *FOXO1/FOXO3/FOXO4* forkhead box O 1/3/4, *GSK3α/GSK-3β* glycogen synthase kinase 3α/β, *USP43* ubiquitin-specific peptidase 43, *USF-1* upstream stimulatory factor-1, *MST-2* mammalian Ste20-like protein kinase-2,* CREB1* CAMP responsive element binding protein, *IKKα* IkappaB kinase α, *YAP1* yes-associated protein 1, *Mdm2* mouse double minute 2 homolog, *ASK1* apoptosis signal-regulating kinase 1, *AR* androgen receptor, *TSC2* tuberous sclerosis complex 2, *4E-BP1* eukaryotic translation initiation factor 4E-binding protein 1, *PRAS40* proline-rich Akt substrate of 40 kDa, *BRCA1* BReast CAncer gene 1, *TRF-1* telomeric repeat factor-1, *GLUT-4* glucose transporter type-4, *GLUT-1* glucose transporter type-1,* TBC1D4* TBC1 Domain Family Member 4, *PIKFYVE* 1-phosphatidylinositol 3-phosphate 5-kinase, *TXNIP* thioredoxin-interacting protein, *TBC1D1* TBC1 Domain Family Member 1, *PFKFB2* 6-phosphofructo-2-kinase/fructose-2,6-biphosphatase 2, *HK-2* hexokinase-2, *ACOT4* acetyl-coA thioesterase-4, *ACLY *ATP citrate lyase, *PDE3B* phosphodiesterase-3B, *eNOS* endothelial nitric oxide synthase

The cessation of Akt activity is essential in suppressing tumorigenesis and is carried out by protein phosphatase 2A (PP2A), the PH domain leucine-rich repeat-containing protein phosphatase 1/2 (PHLPP1/2), which dephosphorylates Akt at the A-loop and HM sites, and the two phosphatases, phosphatase and tensin homolog (PTEN) and Src homology 2 domain-containing inositol-5-phosphatase (SHIP), which convert PIP3 to PI[3],[4] P2 and PI[2, 3] P2, respectively [131–134] (Fig. 1A).

Positive regulation of these negative AKT regulators tilts the balance towards AKT inactivation and vice versa. For example, ERBB receptor feedback inhibitor 1 prevents PHLPP from interacting with AKT [135]. Sirtuin 7 promotes AKT dephosphorylation by PHLPP by deacetylating FK506 binding protein 51 (FKBP51) at lysine residues 28 and 155, which allows PHLPP to form a ternary complex with AKT and FKBP51 [136]. The activity of PP2A is enhanced by the receptor for protein kinase 1 (RACK1), with which it forms a complex [137], as well as aldolase B, which recruits PP2A to phosphorylated AKT [138]. WNK lysine-deficient protein kinase 1 stabilizes PP2A subunits by interacting with protein phosphatase 2 scaffold subunit alpha [139]. AKT can antagonize PP2A via microtubule-associated serine/threonine kinase-like (MASTL) [140]. While inhibitor 1 of PP2A (I1PP2A/ANP32A), inhibitor 2 of PP2A (I2PP2A/SET), and cellular inhibitor of PP2A (CIP2A) can directly associate with and inhibit PP2A [141, 142], the small peptide encoded by the long non-coding RNA LIN00665 can inhibit the activity of CIP2A [143].

Besides dephosphorylation, AKT can be inactivated via SUMO deconjugation (de-SUMOylation), acetylation, and K63-linked ubiquitination, the latter targeting AKT for lysosomal or proteasomal degradation (although it may also activate AKT, as alluded to above). Each of these post-translational modifications is subject to regulation by different proteins. De-SUMOylation can occur in the presence of small ubiquitin-like modifier (SUMO)-specific proteases, SENP 1, 2, and 3 [144]. Proteosome-mediated AKT1 degradation is accomplished by zinc and ring finger 1, tetratricopeptide repeat domain 3, tripartite motif containing 13, and mitochondrial E3 ubiquitin protein ligase 1, which polyubiquitinate AKT1 at K48 [145–148]. K48-ubiquitinated AKT may undergo further ubiquitination at lysines 284 and 214 before being targeted for lysosomal degradation by the arginylated form of HSPA5 (GRP78/BIP) [149]; USP7 opposes the effect of HSPA5 by deubiquitinating AKT at K284 and K214 [148]. The binding of Akt1 to peptidyl-prolyl isomerase Pin1 protects it from proteasomal degradation, which requires phosphorylation of Akt1 at T92/450 [149]. BRCA1-associated protein 1 (BAP1) is a deubiquitinase that, according to some studies, can either stabilize the phosphorylated form of AKT by preventing its ubiquitination in concert with a C-terminally truncated form of mutant additional sex combs-like protein 1 (ASXL1) [150] or inactivate AKT by deubiquitinating and stabilizing PTEN [151]. Acetylation at K14/20 by the histone acetyltransferase P300 and lysine acetyltransferase 2B has been shown to block AKT activation [152]. Finally, AKT can be inactivated by caspase-mediated cleavage during apoptosis [153].

## PI3K-AKT pathway: crosstalk with other pathways

### Crosstalk with the MAPK pathway

The loss of negative feedback and inhibition of the IGF1R, which is normally exerted by phosphorylated ribosomal p70S6 kinase, following treatment with mTOR inhibitors in cancer, and the upregulation of insulin receptor substrate (IRS) 1/2, upon treatment with Akt inhibitors, leading to the activation of the PI3K-Akt and MAPK pathways, hints at the possibility of cross-talk between the two pathways and likely accounts for the reduced efficacy of these drugs [34, 154–159]. Similar upstream receptor tyrosine kinases activate both pathways and often act synergistically to sustain tumorigenicity. Tumors with acquired resistance to tyrosine kinase inhibitors (TKIs) can create a bypass track by increasing the expression of an alternative receptor tyrosine kinase (RTK) that re-activates those very same pathways [160]. Moreover, Ras can activate PI3Kα (and therefore AKT) by gathering PI3K’s substrate, PIP2, and increasing PI3K’s membrane attachment [161].

In other instances, one pathway can compensate for inhibiting another pathway by attempting to return it to its baseline functioning level. For example, long-term treatment with PI3K inhibitors in KRAS-mutant cancer cells can lead to the re-activation of AKT, a process dependent on KRAS’s downstream effector, ERK2 [162]. Decreased clonogenicity of KRAS-mutant cells can be accomplished by combining PI3K inhibitors with MEK inhibitors [162], which likely offsets the proliferative effects of both AKT and ERK. In fact, dual inhibition of AKT and MEK/ERK as a strategy to combat tumors harboring mutant RAS has shown promising results in pre-clinical studies [163, 164]. This is especially relevant since some studies have shown that the MAPK pathway can tone down the production of reactive oxygen species (ROS) generated by the PI3K-AKT pathway, which relies heavily on mitochondrial respiration to meet the anabolic requirements of cancer cells [164]. Conversely, AKT can, in specific settings, downregulate the ERK pathway by phosphorylating c-Raf on T259, effectively deactivating it [99, 165].

### Crosstalk with NF-κB pathway

The NF-κB pathway appears to have a bi-directional relationship with the PI3K-AKT-mTORC1 pathway, especially the EGFR-PI3K-AKT-mTORC1 pathway, intersecting at the level of IKK. NF-κB is usually retained in the cytoplasm by its binding partner, the inhibitor of kappa B (IκB) [166]. It is only when IκB is phosphorylated by IKK and degraded that NF-κB can translocate to the nucleus to activate the transcription of genes involved in cellular proliferation, survival, and angiogenesis, as documented in cases of esophageal cancer [167]. As one of the AKT substrates, IKK can activate the NF-κB pathway and upregulate EGFR expression in a positive feedback loop to enhance the PI3K-AKT-mTORC1 pathway. This makes IKK a desirable target when used as either a sole treatment or in combination with other targeted therapies [168].

### Crosstalk with the Wnt/β-catenin pathway

The Wnt pathway is essential for intestinal homeostasis, where it regulates intestinal stem cell renewal and epithelial cell proliferation, and its overactivation causes cancer [169, 170]. The activation of the Wnt pathway can deactivate Akt signaling, and vice versa, and this has been demonstrated in different cancers [171]. In breast cancer, Nectin-4 indirectly activates the Wnt pathway via the PI3K/Akt pathway, and this, in turn, contributes to tumor maintenance by replenishing the pool of cancer stem cells [172], which is often implicated in treatment failure and tumor relapse. The Wnt pathway can also be ‘switched on’ through the phospholipase PLD1, downregulating ICAT by activating the Akt pathway [171, 173].

### Crosstalk with the JNK and p38 pathways

As the name suggests, upstream kinase apoptosis signal-regulated kinase 1 (ASK1) is an upstream kinase of the JNK and p38 pathways activated by various stress stimuli and induces apoptosis. ASK-1 can be inhibited by AKT, which directly phosphorylates ASK1 on its amino acid residue S83. This, presumably, allows AKT to establish a delicate balance between its pro-tumoral signals and the pro-apoptotic signals of the JNK and p38 pathways [108].

### Crosstalk with Other pathways

**Fig. 2:**
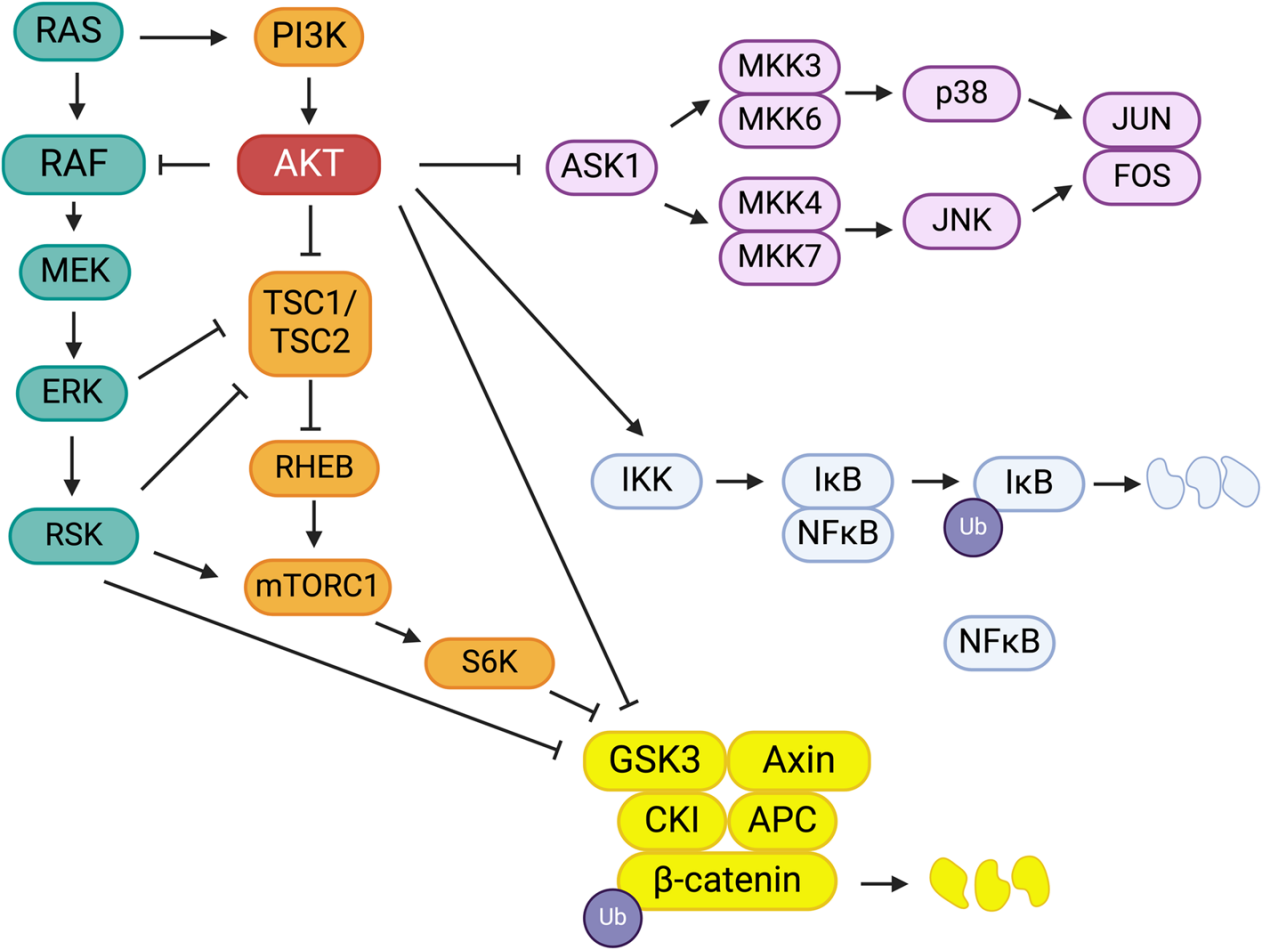
Modular network of crosstalk among AKT and other signaling pathways. Lines with arrow end and blunt end denote functional activation or inhibition, respectively

Rad9, as part of the Rad9-Hus1-Rad1 complex, detects DNA damage and initiates DNA repair by enabling ataxia telangiectasia and Rad3-related (ATR) kinase to phosphorylate its downstream effector, Chk1 [174]. Rad9 is overexpressed in prostate cancer cell lines and clinical samples, where it increases AKT activation and promotes tumor cell migration and anoikis resistance [175]. A non-canonical form of Thr308 phosphorylation and Akt activation involves calcium-calmodulin-dependent kinase, activated by the calcium-calmodulin complex when cytoplasmic calcium levels rise [176]. In melanoma, the increased expression of RUNX2 endows tumor cells with metastatic capability, possibly by re-activating the MAPK and PI3K/AKT pathways [177] Sp1 is a transcription factor whose nuclear translocation is contingent upon its phosphorylation. In cancer cells, Sp1 increases the transcription of genes involved in proliferation, invasion, metastasis, stemness, and chemoresistance [178]. In breast cancer, Sp1 is activated by GDNF via AKT, causing Sp1 to activate, in turn, the ST3GAL1 promoter [179]. Figure 2 illustrates known signaling pathways with which AKT intersects, forming a modular network.

## Mechanisms of AKT deregulation in cancer & overgrowth syndromes

AKT activation in cancer most commonly occurs as a result of amplifications, gain-of-function and loss-of-function mutations, or deletions of AKT pathway genes, including those encoding AKT’s upstream/downstream modulators, such as growth factor receptors (e.g., EGFR), PIK3CA, the p110α catalytic subunit of PI3K, Ras, PTEN, neurofibromin (NF1), serine/threonine kinases (LKB1), and cyclin-dependent kinase inhibitors (p21WAF1 and p27KIP1), and these have been detected in numerous epithelial and hematologic malignancies [7, 180]. Among other genetic alterations involving AKT pathway genes, in acral melanoma, the oncogenic p85β regulatory subunit 2 of PI3K (*PIK3R2*) is amplified along with *PDPK1* (PDK-1), while the tumor suppressor genes *PIK3R1* (encoding p85α) and *PTEN* are lost or mutated [181, 182]. Other mechanisms of AKT activation are discussed below.

### AKT activation by viral (retroviral) infection

As discussed above, the v-*Akt* oncogene was isolated from the AKT8 retrovirus that had originated in an AKR mouse T cell lymphoma [183], but there exists another example of a retrovirus that is a causative agent of mammalian cancer, in part via activation of the AKT pathway. Ovine pulmonary adenocarcinoma (OPA) (in sheep), whose etiological agent is jaagsiekte sheep retrovirus (JSRV), is unique among retroviruses in that it has a tropism for differentiated epithelial cells in the lungs [184]. OPA has been used as an animal model to investigate the molecular underpinnings of pulmonary adenocarcinoma in humans, as it bears striking similarities with its human counterpart, among which is its histological resemblance [185, 186]. Their similarities are also reflected in the type of signaling pathway that is activated, as they both seem to activate the PI3K-AKT pathway. In the case of JSRV, the expression of the envelope protein is enough to transform lung epithelial cells in vitro, which is mediated by the cytoplasmic tail of its transmembrane protein [187]. Surfactant protein A (SPA) regulates surfactant protein B (SPB) secretion via PI3K-AKT-mediated activation of the lung-specific transcription factor, HNF-3β [188, 189]. The long terminal repeat sequences (LTR) in the JSRV genome contain enhancer and promoter elements that, upon entry and integration, are recognized by HNF3β and other members of the host’s transcriptional machinery, which drives the expression of the envelope protein [190, 191]. It is hypothesized that HNF3β is further upregulated via the JSRV-PI3K-AKT axis, creating an autocrine loop that favors JSRV expression in transformed type II pneumocytes [187].

### AKT gene amplification (and overexpression)

*AKT2* was the first AKT gene shown to be recurrently altered in human malignancies, with amplification and overexpression observed in 12–20% of ovarian and pancreatic cancers and cell lines [17, 192–196]. Furthermore, in ovarian cancer, *AKT2* amplification/overexpression is correlated with poor prognosis [193] and high-grade disease [197]. *AKT2* is also overexpressed in approximately 55% of colorectal cancers and nearly 40% of hepatocellular carcinomas [198, 199]. Experimental work showed that overexpression of *AKT2* in ovarian carcinoma cells correlated with increased invasion and metastasis [200]. In vitro kinase assays revealed that more than 30% of pancreatic carcinomas had greater than threefold increased *AKT2* kinase activity compared with normal pancreatic samples and benign pancreatic tumors [201]. Unlike *AKT2*, amplification of *AKT1* is a rare occurrence in human cancer [1, 7, 181, 202]. To our knowledge, amplification of AKT3 has not been reported in any human cancers. However, overexpression of *AKT3* has been reported in about 60% of hepatitis C virus-associated hepatocellular carcinomas [203], 10% of acral melanomas [181], and ~ 20% of ovarian cancers [204].

### Epigenetic modes of AKT activation

#### Activation by RNA methylation

Gene expression is regulated at the post-transcriptional (RNA) level by epi-transcriptomic modifications, of which N^6^-methyladenosine (m6A) is the most abundant type [205]. RNA methylation has a range of effects on RNA splicing [206, 207], nuclear export [208], stability [209, 210], translation [210–212], DNA damage repair [213], initiation of miRNA biogenesis [214], and immunogenicity [215], and can thus affect tumor predisposition and outcomes. Decreased m6A has been reported to impair the translation of PHLPP2 and increase the translation of mTORC2, resulting in AKT hyperactivity [216]; increased methylation and decreased translation of *PTEN* mRNA bring about the same effect in many other cancers [217].

#### AKT activation by microRNAs

Gene expression can also be modulated at the post-transcriptional level by microRNAs (miRNAs). The miRNAs can activate Akt by binding to the 3’ untranslated regions (UTRs) of AKT’s negative regulators, preventing their translation. MAGI-2 is a scaffold protein that recruits PTEN to the plasma membrane and positively regulates its activity, and in some cancers, it is targeted by miR-101 [218]. In hepatocellular carcinoma, miR-21 inhibits the synthesis of *PTEN* mRNA [219], while the overexpression of miR-222, which suppresses the regulatory subunit of protein phosphatase 2A, correlates with advanced-stage hepatocellular carcinoma and shorter disease-free survival [220]. In colorectal carcinoma, *PHLPP2* synthesis is blocked by miR-186-5p and miR-150-5p, eliminating its tumor suppressive effects [221]. Chemoresistance in esophageal cancer is caused by AKT overactivation secondary to miR-200c, downregulating *PPP2R1B*, another regulatory subunit of protein phosphatase 2A [222]. In oral squamous cell carcinoma, elevated levels of miR-182-5p activate AKT by targeting the calcium/calmodulin-dependent protein kinase II inhibitor, CAMK2N1, a potent inhibitor of calcium/calmodulin-dependent kinases II and IV (Ca2 + /CaMK II and Ca2 + /CaMK IV) [223].

#### Long non-coding RNA (lncRNA)-mediated activation

Long non-coding RNAs (lncRNAs) are more than 200 nucleotides long and are not translated into functional proteins. LncRNAs modulate gene expression at the post-transcriptional and transcriptional levels through chromatin modifications and general transcriptional machinery recruitment [224]. The lncRNA H19 and its mature product, miR-675, increase AKT/mTOR signaling in gastric cancer through the lncRNA-H19/miR-675/RUNX1 axis [225, 226], whereas in gallbladder cancer, the same lncRNA can act as an endogenous competing RNA (ecRNA) by decoying miR-294-5p to increase AKT expression [227]. An interesting lncRNA is LINC00470, which forms a ternary complex with the DNA/RNA binding protein, FUS, and AKT in the cytoplasm to increase AKT’s activity, as reported in cases of glioblastoma multiforme; phospho-AKT prevents the ubiquitination of hexokinase-1 (HK-1), thereby increasing glycolysis, inhibiting autophagy, and increasing glioblastoma multiforme’s tumorigenicity [228].

### AKT activation by post-translational modifications

In addition to serine and threonine phosphorylation, Akt is subject to an array of other post-translational modifications, including hydroxylation on proline residues [229–231], methylation [232], phosphorylation on tyrosine residues, O-GlcNAcylation on serine or threonine residues, and SUMOylation, acetylation, and ubiquitination on lysine residues, which can significantly alter AKT activity, even when the function of AKT’s upstream regulators, such as PI3K or PTEN, are unperturbed [233]. The phosphorylation of tyrosine 26 in AKT1 by Mer tyrosine kinase (MERTK) can promote AKT1 activation by the PI3K signaling pathway [234]. Unlike ubiquitination and methylation, it is unclear whether SUMOylation, which is carried out by the SUMO-conjugating enzyme, Ubc9, SUMO-activating enzyme, SAE1, and SUMO E3 ligase, PIAS1 [51, 235, 236], is necessary for AKT phosphorylation [237, 238]; however, as mentioned above, SUMOylation does play a role in enhancing the activity of AKT [50], regardless of PI3K activity and AKT membrane localization [235]. Activated AKT, in turn, can phosphorylate SUMO1 and Ubc9 at T76 and T35, respectively, increasing overall SUMOylation [238]. In papillary thyroid cancer, SIRT7, an NAD + -dependent histone/non-histone deacetylase, may be targetable, as it often shows increased expression; SIRT7 suppresses the transcription of DBC1, an endogenous inhibitor of SIRT1, by deacetylating H3K18Ac. This leads to the deacetylation of AKT and ribosomal p70S6K1 by SIRT1, permitting their phosphorylation and activation [239]. Egl-9 family hypoxia inducible factor-1 (EglN1) is an oxygen sensor that degrades hypoxia inducible factor (HIF) under normoxic conditions. Among the list of EglN1’s substrates is AKT, which hydroxylates at proline residues 125 and 313. AKT is then inactivated by VHL in an E3 ubiquitin ligase-independent manner through dephosphorylation of pThr308 by PPA. Under hypoxic conditions, EglN1 cannot hydroxylate Akt, sending Akt into ‘overdrive’ mode. This may explain how hypoxia fosters treatment resistance in tumor cells [229, 230].

### *AKT* activation by mutations

The vast majority of *AKT1* gene missense mutations encompass the PH domain, the most common one being E17K, which has been reported in bladder, breast, ovarian, endometrial, urothelial, colorectal, lung and pancreatic cancers [240–245]. This mutation is also linked to Proteus Syndrome, a rare disease characterized by overgrowth of skin, bone, and soft tissue, causing significant disfigurement and functional impairment in affected individuals [246]. Activating E17K missense mutations in the *AKT3* gene have been described in acral melanoma, breast, ovarian, and colorectal cancer [181, 247]. E17K enhances AKT’s ubiquitination as well as its affinity for PIP3, substantially increasing its ability to localize to the plasma membrane and be phosphorylated by PDK1 [53, 241]. Other missense mutations, including L52R, C77F, Q79K, and E49K, have also been identified in the PH domain and have similar effects [156, 240]. G171R, present in bladder cancer, affects AKT3’s kinase domain and leads to AKT phosphorylation and hyperactivation as well [248].

### Other factors leading to AKT activation

The non-receptor tyrosine kinase SRC can activate AKT in the presence of oxidative stress [249]. Moreover, protein kinase A (PKA) and exchange proteins directly activated by cAMP (EPAC) can also activate AKT by way of cAMP [104, 250–252]. The effect of heat shock on AKT activation is controversial, with some studies contending that heat shock can activate AKT without the need for PI3K, AKT plasma membrane translocation, and phosphorylation [253] in the case of oligomeric AKT but not monomeric AKT [249]. In contrast, other studies suggest that heat shock aids in AKT phosphorylation and activation [254]. Awareness of these non-canonical modes of AKT activation is essential, as they explain why drugs targeting PI3K/mTOR may not always be effective.

## Role of AKT isoforms in cancer-specific tumor initiation & progression

AKT isoforms may have opposing roles and even protective roles at different stages of tumor progression in different cancers, which is why incorporating therapies that are directed against AKT isoforms is so crucial. This may be due to the differential level of isoform expression in tumors, and this can vary based on the type of tissue from which the cancer arose. Alternatively, different AKT isoform activity may depend on other factors within the PI3K/AKT pathway, such as which upstream PI3K isoforms are activated or the presence of specific gain-of-function PI3K mutations. Other factors include different isoforms having distinct subcellular localizations, different substrate specificities, or varying effects on similar substrates [255]. For instance, AKT2 is selectively activated in the presence of the *PIK3CA* hotspot mutation H1047R [256]. Cancers with *PTEN* loss show increased activity of the PI3K-p110β (PIK3CB) isoform, which preferentially activates AKT2 [257, 258], while cancers with increased activation of SRC-family kinases (SFKs) secondary to RTK stimulation show increased activation of another PI3K isoform, PI3K-p110α (PIK3CA), that activates AKT1 [259].

In transgenic mouse models of mammary carcinoma, driven by Erbb2 and polyomavirus middle T-ag (PyMT), germline *Akt1* gene ablation inhibited primary tumor development and, although increased tumor invasiveness, it did not increase the risk of metastasis. In contrast, the opposite was true when germline *Akt2* was ablated [260]. These findings are significant to note since they emulate drug therapy. In the same Erbb2-driven mammary carcinoma model, expression of activated Akt1 or Akt2, in which the Thr308 and Ser473 residues were replaced with phosphomimetic Asp residues (Akt1 T308D S473D and Akt2 T308D S473D), resulted in increased pulmonary metastases and tumor invasiveness, particularly in the case of activated Akt2 [260–262].

As a proof of concept, Chen et al. compared the effects of cell-autonomous and systemic *Akt1* and *Akt2* deletion on mammary tumorigenesis and found that systemic *Akt2* ablation did not protect against metastasis, while increased primary tumor development was due to a compensatory rise of systemic insulin levels, which hyperactivates Akt and enables ErbB2 activation. They also found that the effect of systemic *Akt2* ablation on primary tumor development can be counteracted by inhibiting insulin. Systemic *Akt1* ablation, on the other hand, blocks metastasis by inhibiting the mobilization and survival of tumor-associated neutrophils, which have pro-metastatic properties; ablating *Akt1* in neutrophils alone is sufficient to impede metastasis [263]. The disconnect between assumed AKT signaling dependency and drug potency is further exemplified by the finding that treatment with the pan-AKT inhibitor, MK2206, in a xenograft model of MCF-7 and MDA-MB-231 human breast cancer cells, increased in vivo lung metastasis, whereas *AKT1* knockdown inhibited the invasiveness of the two xenografts [264].

Using genetically engineered mouse models and tetracycline-regulated *AKT* isoform shRNA, it was found that in prostate cancer, AKT1 promotes tumor growth, and AKT2 promotes metastasis [257, 265]. In a mouse model, knockdown of Akt1 significantly inhibited ovarian cancer cell proliferation and in vivo tumor progression, whereas disruption of Akt2 increased tumor growth [266].

The role of AKT3 in oncogenesis is less clear-cut. Numerous studies have elucidated the role of AKT3 as a driver of endocrine therapy and AKT inhibitor resistance in ErbB2-driven breast cancer and breast cancer in general [267, 268]. In fact, AKT3 is upregulated in ER + breast cancers and androgen-independent prostate cancers, suggesting a role for AKT3 in tumor progression [269]. However, other studies reported decreased migration and metastasis in triple-negative breast cancer cell lines in which AKT3 is overexpressed [270].

## Effect of AKT isoforms on the immune system: implications for inflammatory diseases, cancer prevention and treatment

AKT is essential for regulating T cell development, differentiation, metabolism, and effector function. By phosphorylating FOXO1/3, AKT blocks naturally occurring Treg (nTreg) differentiation of double-positive (DP) thymocytes in the thymus and induces Treg (iTreg) differentiation of antigen-stimulated naïve CD4 + T cells in the periphery and promotes effector CD8 + T cell (versus memory CD8 + T cell) differentiation (reviewed in [271]). However, recent evidence suggests that this is grossly oversimplified, as the fate of CD4 + T cells is governed by the type of substrates that AKT phosphorylates, which is in turn dictated by whether AKT is phosphorylated on Thr308 alone or Thr308 and Ser473, in response to weak or strong TCR stimulation, respectively. Evidence suggests that weak TCR stimulation of CD4 + T cells promotes commitment to the iTreg lineage over other CD4 + subsets [272, 273]. This is substantiated by the fact that ex vivo stimulated human T cells show Thr308 phosphorylation, and expression of a constitutively active AKT in human Treg cells diminishes their suppressive capacity [274]. In response to weak TCR stimulation, AKT favors Treg differentiation by phosphorylating heterogeneous nuclear ribonucleoproteins hnRNP L and hnRNP A1, as confirmed by mass spectrometry-based proteomic analysis; knocking down hnRNP L and hnRNP A1 resulted in a decline in Treg cell number [272]. Weak TCR stimulation via AKT additionally inactivates the citric acid cycle enzyme Citrate Synthase, allowing acetyl CoA to be instead used for the decompaction of chromatin at the *FOXOP3* promoter to promote FOXOP3 expression and iTreg differentiation[275].

Given that PD-1 blockade can expand the number of intratumoral memory T cells [276], and given AKT’s implication in PD-L1 upregulation in some tumors [277], combining AKT inhibitors with anti-PD-1/PD-L1 therapy can produce robust anti-tumoral responses to maximize therapeutic efficacy [278–280]. Pharmacological manipulation with AKT inhibitors of tumor-infiltrating lymphocytes (TILs) isolated from cancer patients reprogrammed them into acquiring a stem-like memory cell phenotype, which increased their life span when transferred into NOD scid gamma (NSG) mice [281]. Ex vivo treatment with AKT inhibitors of cytotoxic T lymphocytes (CTLs) isolated from a mouse model of melanoma, and CAR-T cells in a murine leukemia xenograft model, and their re-administration to the mice produced similar results, with better tumor control and improved overall survival in both cases [281, 282]. One study, however, contradicted these findings and maintained that the overexpression of AKT in tumor-specific T cells results in superior outcomes [283]. It was also found that inhibition of Akt1 and Akt2, but not Akt3, decreases terminal CD8 + T cell differentiation, suggesting that Akt isoforms differentially regulate CD8 + T cell differentiation in the same way they regulate Treg differentiation [284] (see below).

As in tumor cells, AKT isoforms may act in opposition to regulate Treg cell differentiation. The genetic ablation of *Akt1* relieved T cell-mediated CNS dysfunction in a murine model of experimental autoimmune encephalomyelitis [285]. In contrast, in another study, the genetic ablation of Akt2 and Akt3 had the opposite effect, suggesting that Akt1 blocks FOXO1-mediated FOXOP3 induction and inducible Treg (iTreg) differentiation in this setting [286]. However, another study contradicted these findings and concluded that the Akt2 isoform, not the Akt1 isoform, limits iTreg differentiation [287]. Human Treg cells are similar to mouse Tregs in that AKT3, but not AKT2, appears to direct CD4 + T cells toward iTreg differentiation, and they lose suppressive functions and adopt a Th1 profile in the presence of AKT1 [288].

The observation that Akt isoforms have opposing effects on tumorigenesis and Treg differentiation can be extended to macrophages. Macrophages present in the tumor microenvironment (TME) that acquire an M2 phenotype can promote tumor progression and metastasis by secreting immunosuppressive cytokines, such as transforming growth factor beta (TGFβ) and interleukin-10 (IL-10) [289, 290], increasing angiogenesis [291], and remodeling the stroma by producing matrix metalloproteinases (MMPs) [292]. As a result, recent efforts have been directed towards targeting M2 macrophages or attempting to revert them to an M1 phenotype, which, in contrast to M2 macrophages, is known to be tumoricidal and pro-inflammatory [293].

In the absence of ICAM-1, a transmembrane glycoprotein belonging to the immunoglobulin superfamily, macrophages acquire an M2 phenotype in the presence of apoptotic tumor cells through efferocytosis, as shown by co-culture experiments, and this is mediated by AKT, which upregulates M2 genes; the systemic knockout of the *ICAM1* gene increased the development of liver metastasis in a mouse model of colon cancer compared to *ICAM1* wild-type littermates [294].

Akt2 increased the chemotaxis of mouse peritoneal macrophages and THP-1 cells in response to the tumoral chemotactic factor, CSF-1, by increasing LIMK/Cofilin phosphorylation and actin polymerization, which was abolished by knocking down *Akt2* using small RNA interference (siRNA) [295]. While Akt1 increased M1 macrophage polarization by positively regulating miR-155 [296], myeloid-specific ablation of miR-155 in a murine model of spontaneous mammary carcinogenesis accelerated tumor growth by increasing M2 macrophage polarization [297]. In the liver, hepatocellular carcinoma developing in *Akt2* knockout mice after hepatic *Akt1* ablation showed increased infiltration of macrophages expressing Akt1 [298], which may indicate that Akt1 polarizes macrophages towards the M2 phenotype.

In a model of dextran sodium sulfate (DSS)-induced colitis, exacerbation of intestinal inflammation occurs when *Akt1* is ablated due to macrophages acquiring an M1 phenotype, whereas when *Akt2* is ablated, macrophages acquire an M2 phenotype and the inflammation remits, suggesting that Akt2 could potentially be targeted to both treat colitis and prevent colitis-associated neoplasia. It should be noted, however, that in this study, macrophage depletion and reconstitution experiments confirmed that the lack of Akt activity in other cells could also contribute to the exacerbation of DSS-induced colitis and that, in addition to macrophages, these cells may play a role in the pathogenesis of inflammatory bowel disease (IBD) in humans [299].

The unique interplay between Akt1, Akt2, and Akt3 in hepatic stellate cells (HSCs), Kupffer cells, and hepatocytes in mediating inflammation, cell proliferation, migration, and fibrogenesis has also been implicated in alcoholic liver disease (ALD) progression, which was revealed in lipopolysaccharide (LPS)- and ethanol-induced two-hit model of ALD, both in vitro and in vivo. Cell culture experiments showed that siRNA-directed silencing of *Akt2* downregulated inflammatory markers in HSC and Kupffer cells and that both Akt1 and Akt2 inhibited cell proliferation and fibrogenesis in hepatocytes and HSCs, but only Akt2 inhibited cell migration. Treating mice with a pharmacological agent that blocks Akt2 suppressed binge ethanol and LPS (EBL)-induced inflammation, whereas Akt1 and Akt2 blockers downregulated pro-fibrogenic gene expression and halted the progression of fibrosis [300].

## AKT inhibitors: clinical trials & current therapeutic challenges

Four categories of drugs have been used to target AKT: 1) those that compete with ATP for binding to the active site of AKT (competitive AKT inhibitors) and stabilize the active conformation of AKT; 2) those that bind to the molecular interface of the PH and kinase domains, and stabilize the inactive “PH-in” conformation of AKT (allosteric AKT inhibitors) [301]; 3) PIP3 analogues, which bind to the PIP3-cavity within the PH domain [156]; and 4) the newer generation covalent-allosteric AKT inhibitors (CAAIs), in which allosteric inhibition is combined with the irreversible covalent modification of the two cysteine residues in AKT’s activation loop, translating to a prolonged target occupation time [302].

**Fig. 3:**
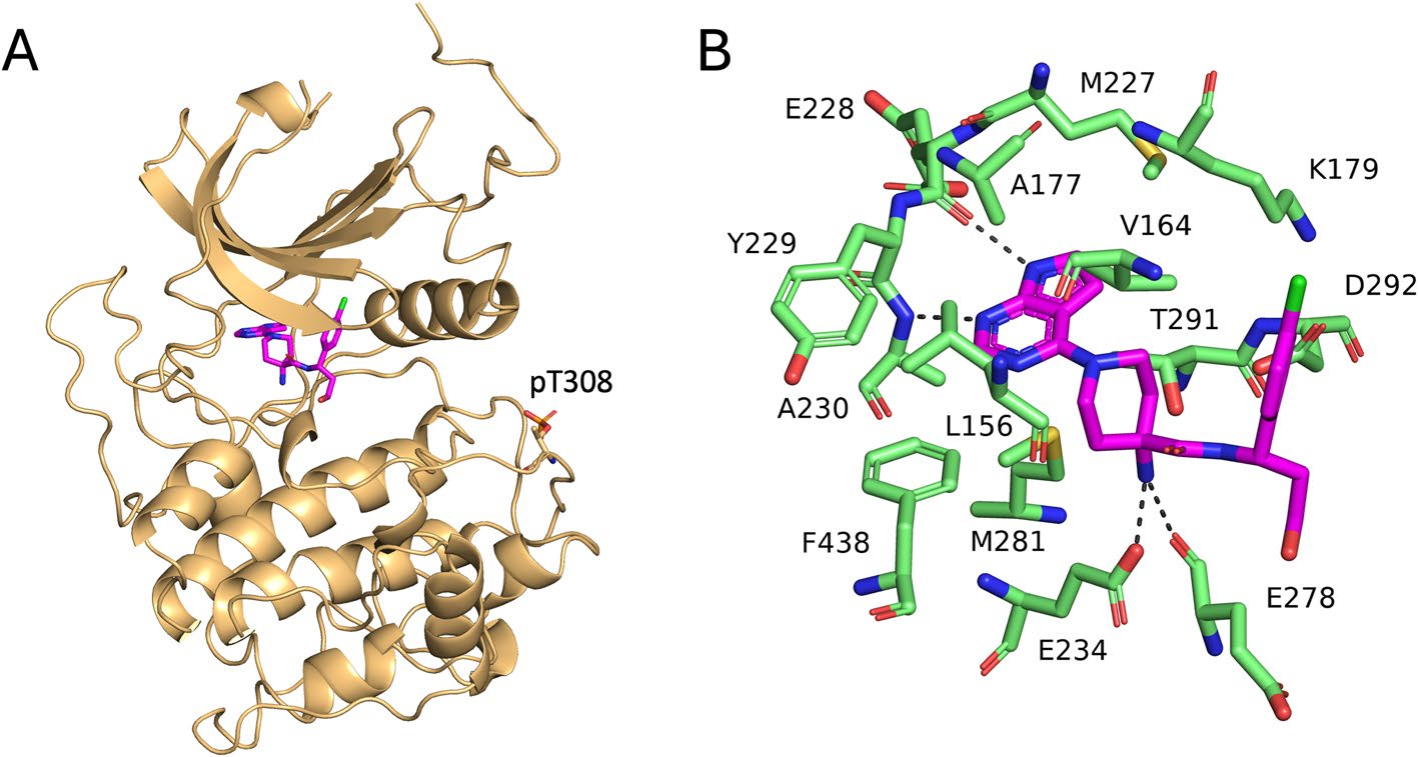
Structure of the complex of human AKT1 with capivasertib (PDB entry 4GV1). **A** Schematic of the complex of AKT1 (gold) with capivasertib (purple); phosphorylated Thr308 on activation loop is shown in sticks. **B** Close-up of AKT1 interacting residues that are within 4 Å of capivasertib. Dotted lines indicate hydrogen bonds

Modified PIP3 analogs suffer from poor drug-like properties and selectivity due to the presence of other molecules within cells that contain structurally related PH domains [303]. The ATP-competitive inhibitors capivasertib (AZD-5363) and ipatasertib (GDC-0068), which have recently progressed to phase III in clinical trials for the treatment of hormone receptor (HR)-positive, HER2-negative breast cancer, and triple-negative breast cancer, in combination with fulvestrant (CAPItello-291), the CDK4/6 inhibitor palbociclib (CAPItello-292), and paclitaxel (CAPItello-290) [304], also suffer from lack of specificity, as the ATP-binding pocket is conserved among kinases in human cells, and the clinically observed decrease in efficacy is often due to dose reduction in an attempt to counter toxicity [156]. A list of published, completed clinical trials of AKT inhibitors, including capivasertib, in breast cancer, can be found in Table 2. The structure of the complex of human AKT1 with capivasertib is shown in Fig. 3.

**Table 2:** Completed Clinical Trials of AKT Inhibitors in Breast Cancer

**AKT inhibitor**	**Trial name**	**Phase**	**Study arm**	**Study population (n. enrolled)**	**Study design**	**Primary endpoint**	**Efficacy outcome**	**Ref.**
**Capivasertib**	STAKT	0 (WoO)	Capivasertib or placebo	Early ER + BC (neoadjuvant) (n. 48)	Randomized, double-blind	Changes in AKT pathway markers	NA	[305]
	D3610C00001	I	Capivasertib monotherapy	PIK3CA-mut ER + mBC (part Cb) (n. 31)	Multipart, open label	Safety	Tumor shrinkage: 46% ORR: 4%	[306]
	D3610C00001	I	Capivasertib +/- Fulvestrant	AKT1^E17K^ mut ER + mBC (part D) (n. 63)	Multipart, open label	Safety	ORR (monotherapy): 20% ORR (combination prior fulv.): 36% ORR (combination fulv. Naïve): 20%	[307]
	FAKTION	Ib/II	Capivasertib or placebo + fulvestrant	ER + HER2- mBC, postmenopausal (n. 140)	Randomized, double-blind	PFS	mPFS: 10.3 (capiv) vs 4.8 (pbo)	[308]
	BEECH	Ib/II	Capivasertib or placebo + Paclitaxel	ER + HER2 – mBC (n. 110)	Randomized, double-blind	PFS in ITT and PIK3CA-mut pop	mPFS ITT: 10.9 (capiv) vs. 8.4 (pbo) months mPFS PIK3CA-mut: 10.9 (capiv) vs 10.8 (pbo) months	[309]
	PAKT	II	Capivasertib or placebo + paclitaxel	mTNBC (n. 140)	Randomized, double-blind	PFS	mPFS: 5.9 (capiv) vs. 4.2 (pbo) months	[310]
**Ipatasertib**	FAIRLANE	II	Ipatasertib or placebo + paclitaxel	Early TNBC (neoadjuvant) (n. 151)	Randomized, double-blind	pCR in ITT and PTEN-low popul	pCR ITT: 17% (ipat) vs 13% (pbo)	[311]
							pCR PTEN-low: 16% (ipat) vs. 13% (pbo)	
	LOTUS	II	Ipatasertib or placebo + paclitaxel	mTNBC (n.124)	Randomized, double-blind	PFS in ITT and PTEN-low popul	mPFS ITT: 6.2 (ipat) vs 4.9 (pbo) months	[312]
							mPFS PTEN-low: 6.2 (ipat) vs. 3.7 (pbo) months NA	
**MK-2206 **	NA	0 (WoO)	MK-2206 monotherapy	Early BC (neoadjuvant) (n. 12)	Open label, single arm	pAKT reduction in tumor tissue	NA	[313]
	SU2C	Ib	MK-2206 + paclitaxel	mBC (expansion cohort) (n. 13)	Open label dose finding	MTD	ORR: 23% CBR: 46%	[314]
	NA	I	MK-2206 + anastrozole and/or fulvestrant	ER + HER2 – mBC (n. 31)	Open label dose finding	RP2D	CBR: 36.7%	[315]
	NA	I	MK-2206 + trastuzumab	HER2 + mBC^a^ (n. 27)	Open label dose finding	MTD/RP2D	ORR: 7.4% CBR: 22%	[316]
	NA	I	MK-2206 +/- Lapatinib	HER2 + mBC (escalation + expansion cohort) (n. 8)	Open label dose finding	MTD/RP2D	ORR: 0%	[317]
	NA	Ib	MK-2206 + paclitaxel + trastuzumab	HER2 + mBC (n. 12)	Open label dose finding	RP2D	ORR: 75%	[318]
	NA	II	MK-2206 Monotherapy	PIK3CA/AKT mut or PTEN altered mBC (n. 27)	Open label single arm	ORR	ORR PIK3CA/AKT mut: 5.6%	[319]
	NA	II	MK-2206 + anastrozole	PIK3CA-mut ER + HER2 – early BC (n. 16)	Open label single arm	pCR	ORR PTEN altered: 0% pCR rate: 0%	[320]
	I-SPY2	II	MK-2206 + standard NAT or standard NAT	Early BC (neoadjuvant) (n. 352)	Open label randomized adaptive	pCR	pCR e-rate overall: 35% (exp) vs. 21% (contr) pCR e-rate (ER+/HER2-): 17% (exp) vs. 13% (contr)pCR e-rate (ER-/HER2+): 62% (exp) vs. 35% (contr)	[321]

*Note*: Adapted from [447]

*Legend*: *AC* doxorubicin and cyclophosphamide, *BC* breast cancer, *Capiv* capivasertib, *CBR* clinical benefit rate, *Contr* control arm, *ER* estrogen receptor, *E-rate* estimated-rate, *Esp* experimental arm, *Fulv* fulvestrant, *HR* hazard ratio, *HT* hormone therapy, *Ipat* ipatasertib, *ITT* intention-to-treat, *m* metastatic, *mPFS* median progression-free survival, *MTD* maximum tolerated dose, *Mut* mutated, *NA* not applicable, *NAT* neoadjuvant therapy, *ORR* objective response rate, *Pbo* placebo, *pCR* pathologic complete response, *Popul* population, *RP2D* recommended phase II dose, *TNBC* triple-negative breast cancer, *WoO* window of opportunity

^a^These trials also enrolled patients with HER2+ advanced gastric cancer. However, only results about BC patients are reported

To decrease side effects associated with ATP-competitive inhibitors, allosteric (PH-domain) pan-AKT inhibitors, such as MK-2206, miransertib (MK-7075), and its next-generation inhibitor, Arq751, were developed; both miransertib and Arq571 are currently under investigation for the treatment of Proteus syndrome [322, 323], with positive results being reported for miransertib, based on the results of a 5-year follow-up phase I pharmacodynamic study of an 18-year-old who derived significant benefit from the drug, permitting continued use of miransertib to assess its long-term safety profile [324]. The CAAI borussertib, despite being more efficacious compared with other AKT inhibitors, and despite showing anti-proliferative effects in cancer cell lines harboring alterations of the PI3K/AKT pathway, as well as in a *KRAS*-mutant xenograft model in combination with a MEK inhibitor, has a poor pharmacokinetic profile, making it difficult to achieve an effective therapeutic dose with oral application [302, 325]. Ongoing clinical trials of AKT inhibitors for cancer therapy are listed in Table 3.

**Table 3:** Completed & ongoing clinical trials of AKT inhibitors for cancer therapy as of August 2024

Drug	Company	Alternative names	Drug Class	Targets	Trial phase	ClinicalTrials.gov Identifier
MK-2206	Merk & Co	MK-2206 hydrochloride	Allosteric	AKT 1/2/3	II I I II I	NCT01333475 NCT01480154 NCT01344031 NCT01294306 NCT01245205
GSK2110183	GlaxoSmithKline	Afuresertib	ATP-Competitive	AKT 1/2/3	I/II	NCT01476137
GSK2141795	GlaxoSmithKline	Uprosertib	ATP-Competitive	AKT 1/2/3	I II I I/II II II II	NCT01138085 NCT01941927 NCT01935973 NCT01902173 NCT01964924 NCT01989598 NCT01979523
AZD5363	AstraZeneca	Capivasertib	ATP-Competitive	AKT 1/2/3	II Ib/II I II II II III	NCT02523014 NCT02208375 NCT02338622 NCT02117167 NCT05593497 NCT02299999 NCT03903835
GDC-0068	AbbVie	Ipatasertib	ATP-Competitive	AKT 1/2/3	I II II Ib II II I/Ib Ib/II II Ib II II II II II	NCT03959891 NCT06400251 NCT05554380 NCT04253561 NCT02301988 NCT04551521 NCT05172245 NCT05538897 NCT05332561 NCT01562275 NCT03395899 NCT02162719 NCT01896531 NCT05564377 NCT02465060

As AKT plays a critical role in normal cell physiology, particularly in glucose homeostasis, off-target effects continue to be problematic, even with CAAIs and allosteric inhibitor treatment; diarrhea, hyperglycemia, and liver injury with elevation of liver enzymes were among the side effects observed in many clinical trials [316, 326–330]. The hyperinsulinemia resulting from pan-AKT inhibition can decrease the efficacy of these drugs, as alluded to previously. As the deletion of *Akt1* and *Akt2* genes in hepatocytes results in liver damage, hepatocyte death, inflammation, and the secretion of inflammatory cytokines, including IL-6, leading to STAT3 activation in surviving hepatocytes, which can potentially lead to their transformation [298], it is ill-advised to treat obese patients or those with pre-existing liver injury with pan-AKT inhibitors, as these groups of patients may be prone to developing hepatocellular carcinoma (HCC) if liver injury is sustained. Moreover, treating HCC with pan-AKT inhibitors could prove futile for similar reasons [331].

Most, if not all, of these pan-AKT inhibitors, have failed to progress to phase III as monotherapies, highlighting the need for combining AKT inhibitors with other treatments due to the complexity of AKT biology, with tumor cells are possibly adopting alternative signaling circuitries through feedback loops, downstream target alteration, de novo resistance through loss of negative feedback inhibition (discussed previously), and cross-talk between different pathways [301, 329]. Resistance to AKT inhibitors in breast cancer, for example, can be caused by TSC1/2 loss, which activates mTORC1 and blocks apoptosis in a BAK-dependent manner, even with a reduced level of phosphorylated AKT, possibly by mTORC1-mediated translational control of Mcl-1, and can be overcome by combining AKT inhibitors with an Mcl-1 inhibitor [332, 333]. Moreover, the PI3K-AKT pathway has been implicated in resistance to chemo- and radiotherapeutic agents [334], necessitating the combination of endocrine therapy, targeted therapy, or chemoradiation with AKT inhibitors (reviewed in [335]). MERIT40, for example, is a component of the BRCA1-A DNA repair complex, which undergoes phosphorylation and activation by AKT in response to doxorubicin treatment and promotes resolution of chemotherapy-induced DNA damage [336]. In HER2 + breast cancer cell lines, resistance to anti-HER2 monotherapy is associated with PIK3CA mutations, leading to continuous PI3K-AKT signaling [337]. Furthermore, AKT-independent, PI3K-dependent cancer progression pathways exist [338, 339], meaning that additional drugs targeting multiple nodes upstream of AKT, such as multiple PI3K isoforms and receptor tyrosine kinases, might be required.

Another problem with these pan-AKT inhibitors is that there is a need for complete understanding as to how they exert their effects since some have been shown to inhibit one isoform over another preferentially. As an example, GSK2142795 inhibits AKT2 more potently than AKT1 or AKT3, and another pan-Akt inhibitor, GSK2110183, showed more potent inhibition of AKT1, using in vitro kinase assays containing purified AKT1, AKT2, AKT3, and a GSK3α peptide substrate. Moreover, cancer cell lines harboring PTEN loss or mutant *PIK3CA* required a higher drug concentration to establish 50% growth inhibition (IC50) in 2D cultures compared to that needed to inhibit AKT kinase activity [340].

Lastly, aside from the AKT1-specific inhibitor A-674563 and the AKT2-specific inhibitor CCT128930 (both ATP-competitive inhibitors), most AKT inhibitors lack isoform specificity, and isoform-specific treatments should be tailored to the cancer in question for reasons mentioned above. Even with A-674563 and CCT128930, which purportedly also inhibit PKA and CDK2 [341], no in vitro kinase assays to test their isoform preferentiality have been performed to date [27]. Like pan-AKT inhibitors, it is not entirely clear what the mechanism of action of A-674563 in tumor cells is since it increased (PRAS40) or had no effect (GSK3β) on the phosphorylation of substrates shared by all AKT isoforms, although it is expectedly decreased the phosphorylation of FOXO1 [342].

Despite all of this, capivasertib has shown immense promise for the treatment of breast cancer, with positive results also being achieved in patients with Cowden syndrome [343], who inherit a defective *PTEN* gene in the germline and carry an 85% cumulative risk of developing breast cancer in their lifetime [42]. Capivasertib showed pre-clinical efficacy when used as a single agent for treating human breast cancer cell lines with alterations in *PIK3CA* and *MTOR*, and more so when combined with anti-HER2 and endocrine therapy [304]. In phase I clinical trials of metastatic, estrogen receptor (ER)-positive, HER2-negative breast cancers harboring PTEN loss-of-function and *AKT1* E17K mutations, capivasertib plus fulvestrant was shown to be more tolerable and clinically effective than treatment with capivasertib alone, especially in fulvestrant pre-treated patients, including those who have a history of progression on fulvestrant, with most of the ≥ grade 3 adverse effects reported being diarrhea (5% vs. 10%), hyperglycemia (5% vs. 30%), and a rash (9% vs. 20%) [307, 344]. Similar encouraging results were obtained in both phase I and II trials of HR-positive and HER2-negative breast cancer when capivasertib was co-administered with paclitaxel or olaparib [304].

The PI3K/AKT pathway is also altered in gynecological malignancies. For example, genetic abnormalities of the PI3K/AKT pathway are frequently observed in primary ovarian cancer and predict patient outcomes [345, 346]. Thus, several attempts have been made to target the pathway in these cancers, with promising results [347, 348]. In particular, capivasertib, in combination with olaparib in a phase Ib dose expansion trial, demonstrated durable activity, especially in endometrial cancer. Of the 19% of patients with recurrent triple-negative breast, ovarian, fallopian tube, or peritoneal cancer who partially responded to the treatment regimen, those with endometrial cancer derived the most significant benefit and had the highest partial response (PR) rate (44.4%) [349].

In gastric cancer (GC), increased AKT kinase activity is associated with a higher tumor grade and a poorer prognosis [350] and is observed in up to 78% of tumors [351]. Moreover, mesenchymal-type gastric cancer cell lines were found to be sensitive to agents targeting the PI3K/AKT/mTOR pathway [352], suggesting that GC can be targeted with AKT inhibitors. Data obtained from phase II studies of AKT inhibitors in molecularly selected GC patients found limited clinical benefit, however, along with significant toxicities [353], although, in the umbrella VIKTORY (targeted agent eValuation In gastric cancer basket KORea) trial, which classified metastatic gastric cancer patients based on the presence of 10 different biomarkers and assigned patients with *PIK3CA* mutations and wild-type *PIK3CA* to combination therapy with capivasertib and paclitaxel, the treatment arm with *PIK3CA* mutations derived significant anti-tumor benefit, with an ORR of 33.3% in second-line GC, compared to the low response rate (< 15%) in the *PIK3CA* wild-type group [354]. This suggests that optimization of the therapeutic efficacy of AKT inhibitors in GC can be attained using a biomarker-based approach, which will require further investigation in additional phase II/III clinical trials.

In prostate cancer, however, capivasertib has yielded inconclusive results. In the randomized, placebo-controlled, phase II ProCAID trial of metastatic, castration-resistant prostate cancer, the addition of capivasertib to docetaxel and prednisolone resulted in a statistically significant improvement in median overall survival (OS) of 31.15 months compared to docetaxel and prednisolone alone (20.27 months). However, no statistical significance was reached in composite progression-free survival (cPFS) (7.03 months in the capivasertib group vs. 6.70 months in the placebo group), a primary endpoint that included prostate-specific antigen (PSA) progression. The observed OS result in the capivasertib plus placebo group will need to be validated in prospective studies to address the potential for bias [355].

## Increasing the therapeutic window of AKT inhibitors: future challenges and novel approaches to targeting AKT

Impaired glucose tolerance resulting from pan-AKT inhibition can be overcome by adding metformin treatment regimens, especially since metformin has anti-oncogenic effects, based on the results of prior studies [356]. Recently, the cholesterol-lowering drug pitavastatin was shown to synergize with AKT inhibitors in killing of triple-negative breast cancer cell lines, organoids and xenografts, but not ER-positive cell lines and organoids [357]. While it may be possible to correct faulty genes involving the PI3K-AKT pathway through the use of chimeric genome editing tools, such as Clustered Regularly Interspaced Palindromic Repeats (CRISPR), Transcription-like effector nucleases (TALENs), and zinc-finger nucleases (ZFN) (reviewed in [358]), these tools are, for the most part, restricted to research settings, and they are only mentioned here for the sake of completeness.

Newer approaches developed for targeted protein degradation (TPD) to date have taken advantage of the ubiquitin-proteosome system (UPS) and autophagy/lysosome degradation systems present in eukaryotic cells, the list of which includes PROTACs [359, 360], molecular glues[361, 362], Trim-Away [362], tag-targeted protein degraders [363], specific and non-genetic inhibitors of apoptosis protein-dependent protein erosive agents (SNIPERs) [364], autophagy-targeting chimeras (AUTACs) [365], lysosome-targeting chimeras (LYTACs) [366], and autophagosome tethering compounds (ATTECs) [367]. The benefits of using PROTACs far surpass those of traditional AKT inhibitors: heterobifunctional degraders tend to exhibit significantly prolonged effects compared with AKT inhibitors, as their pharmacological effects depend on the re-synthesis rate of the protein of interest and not target occupancy. INY-03–041 is a pan-AKT degrader composed of the ATP-competitive AKT inhibitor, GDC-0068, conjugated to an E3 ubiquitin ligase substrate adaptor recruiter; INY-03–041 was demonstrated to have significantly prolonged effects on downstream signaling and enhanced potency, which may explain its superior anti-proliferative effects [368]. The translation of the above drugs to the clinic, however, has been hampered by their poor solubility, non-specificity of their biodistribution, off-target systemic toxicity, difficulty finding suitable ligands for the protein of interest [369], as well as their large molecular weights, which impede their cell membrane traversal and concentration in tissues, resulting in reduced target occupancy [370].

A non-exhaustive list of oligonucleotide-based therapeutics includes RNA interference (RNAi) (miRNA mimics, shRNA, siRNA. piRNA) [371], anti-sense oligonucleotides (ASOs) (anti-miRNA oligonucleotides, peptide nucleic acids, Locked Nucleic Acid (LNA), morpholinos) [372], ribozymes [373], long non-coding RNAs (LncRNA) [374], and CRISPR [375], which are all designed to bind to target RNA transcripts via complementary base-pairing. While it is relatively non-cumbersome to construct sequences that match a target of interest with variable specificity, these modalities, like PROTACs, suffer from many drawbacks, including immunogenicity [376], instability imparted by their 2’ hydroxyl (OH) groups [377], toxicity arising from tissue non-selectivity (except for the liver and kidney) and the platform used for drug delivery, as well as poor tissue uptake and endosomal escape [378]. This, combined with the fact that some RNA regions form intricate secondary and tertiary structures often needed for their processing and function, makes the base-pairing design less efficient for the target RNA binding [379]. To overcome these issues, a wholly new and specific approach to targeting RNA, the ribonuclease-targeting chimeras (RIBOTACs) came to the forefront, fusing small molecules with RNA binding ability to a 2'–5'-linked tetra-adenylate conjugate, similar to oligoadenylates produced by cells in response to a viral infection, for RNAse L recruitment, thus converting any inert RNA-binding small molecule into a bioactive RNA degrader, i.e., the RIBOTAC [380]. The ability of RIBOTACs to degrade multiple target RNAs in succession, a feature it shares with PROTACs, means that only low concentrations are required to achieve phenotypic effects, giving RIBOTACs an advantage over oligonucleotide-based therapeutics [381]. However, only 50–60% of the target RNA has been reported to be degraded at any given point in time, possibly due to the rapid turnover of the target RNA. This attribute is intrinsic to RNA species in general rather than a problem with RIBOTACs specifically [382]. As with PROTACs, these molecules’ high molecular weight and charged nature give them inferior physicochemical properties [383]. Another disadvantage inherent to RIBOTACs is the difficulty in finding small molecules that bind selectively to the RNA molecule of interest [381]. Furthermore, the RNA-binding and RNAse-recruiting ligands must be oriented so that RNAse L and the target RNA can interact, which is challenging to accomplish on a spatial level. Yet another problem with RIBOTACs that may be overlooked is that they do not work equally well in all cells since RNAse L expression levels vary among different tissue types [382]. Proximity-induced nucleic acid degraders (PINAD), which have been successfully used to target structural genomic variants of SARS-CoV-2, represent a ‘new and improved’ version of RIBOTACs, wherein the RNAse recruiting ligand is replaced by an imidazole group, a component of the active site of many ribonucleases [382]. It can be envisioned that both RIBOTACs and PINADs could be directed onto *AKT* mRNAs as a future option for therapy. Even positive upstream and downstream AKT regulator transcripts, and oncogenic non-coding RNAs, such as miRNAs and lncRNAs, that target negative AKT regulators and show altered activity or levels in various cancers, as described in earlier sections of this review, could be targeted with such RNA-based modalities. By using CRISPR activation (CRISPRa) (reviewed in [384]) and genetically engineering long non-coding RNAs (lncRNAs) [374], it is even possible to promote the transcription and translation of suppressors of the AKT pathway. This is especially true if the mechanism of their inactivation is epigenetic in origin.

Honing in on each AKT isoform individually, akin to some of the CAAIs that have been in recent development [385, 386], by potentially targeting sequences or residues at the transcript or protein level, that are unique to each isoform, using the above approaches, would yield more desirable outcomes. Whereas these can target the isoforms at the post-transcriptional/translational and post-translational levels, CRISPR interference (CRISPRi) [387] and lncRNAs [374], for example, can repress the respective genes at the transcriptional level. Still, before this can be undertaken, one must attempt to clarify the relative expression levels of each AKT isoform in various cancer types, as well as dive deeper into what roles they play in different cancer progression ‘parameters’. The latter goal can be attained by identifying the substrates of each isoform using cellular proteomic analysis of peptides performed on various cancer cell lines by either knocking out isoform-specific genes, silencing them using siRNA technology [27, 388], or inhibiting each isoform using the recently developed isoform-specific nanobodies [389] or CAAIs [386]; the latter three methods can also form the basis for the development of isoform-specific drugs, for example, isoform-specific anti-sense oligonucleotides (ASOs). The AKT2-specific nanobody, Nb8, targets the hydrophobic motif and was found to induce cell cycle arrest, autophagy, and the loss of focal adhesions in MDA-MB-231 cells by reducing hydrophobic motif phosphorylation [390]. The problem with identifying AKT isoform substrates using cellular proteomics, however, is its inability to distinguish between AKT and non-AKT substrates; for example, other kinases, such as Proviral Integration site for Moloney murine leukemia virus 2 (PIM2), ribosomal S6 kinase (RSK), or PKA, recognize similar versions of the AKT substrate motif [245, 391–393]; PIM2 phosphorylates similar sites on the anti-apoptotic protein and cell cycle regulator, BAD and p21WAF, as AKT, and S455 on ATP citrate lyase (ACLY) can additionally be phosphorylated by PKA, mTOR, or Branched-Chain Ketoacid Dehydrogenase Kinase (BCKDK) [394–399].

Another problem with cell-based assays is that they can confound the results because of compensation by other AKT isoforms [32]. This problem can be overcome by performing LC–MS/MS on phospho-serine and -threonine peptides following the re-expression of each AKT isoform in Akt1/2/3 knockout lung fibroblasts generated from transgenic mice; this approach identified IWS1, among other substrates, as being an Akt1- and Akt3-preferred substrate [400]. One of the limitations of this approach is that the findings only apply to a single cell type (fibroblasts), and they do not factor in non-canonical substrate motifs recognized by each isoform.

Yet a third issue is assigning substrates to a particular isoform when, under specific cellular conditions or in certain cell types, only one isoform is expressed [27]. As a case in point, EZRIN was initially identified as an AKT2 substrate in Caco-2 cells, even though AKT1 and AKT3 are not known to be expressed at sufficiently high levels in these cells [401]. Likewise, the identification of AKT isoform substrates using in vitro assays has the disadvantage of lacking cellular compartmentalization, meaning that substrates that may not interact with specific AKT isoforms within cells may be falsely labeled as being an isoform’s substrate by interacting with said isoform in vitro. Hence, identifying substrates shared by and unique to each AKT isoform requires the integration of results obtained from both in vitro and cell-based assays [27].

On the other hand, isoform compensation might be a problem encountered in the application of isoform-specific therapy [32, 402, 403], which is why targeting more than one isoform might be a more effective therapeutic strategy. Although pan-AKT inhibitors can theoretically negate these effects, they are not without problems, as previously discussed.

Aptamers are short DNA- or RNA-based oligonucleotides that, upon folding into unique secondary and tertiary conformations, can recognize different target molecules, such as metal ions, proteins, protein aggregates and metabolites [404]. Aptamers are equivalent to antibodies in terms of their affinities and specificities for target molecules, but are easier to synthesize and modify, are inexpensive, do not elicit an immune response, can self-assemble, and have the ability to switch conformations with ease [405]. The inherent weakness of PROTACs have resulted in the emergence of two targeted protein strategies that combine PROTACs with aptamers, called aptamer-PROTAC conjugates [369] and aptamer-based PROTACs [406]. However, the use of aptamers for targeted protein degradation has many downsides [369, 404], many of which can be successfully navigated through the use of nanoparticles, notably lipid nanoparticles, which have shown success in clinical trials as delivery vessels for genetic material-containing drugs [407]. Table 4 summarizes the main protein-based modalities that have been employed for targeting the PI3K-AKT pathway, and their mechanisms of action.

**Table 4:** Protein-based approaches for targeting the PI3K-AKT pathway

Approach	Mechanism(s) of Action	Examples of Applications	Ref
PROTACs	A linker connects an E3 ligand to a POI ligand. The POI ligand binds to the POI, and the E3 ligand recruits E3 ligases to the POI to ubiquitinate and mark the POI for degradation by the proteosome system.	Development of INY-03-041, a pan-AKT degrader derived from the ATP-competitive AKT inhibitor, GDC-0068, conjugated to lenalidomide, which recruits the E3 ubiquitin ligase substrate adaptor, CRBN.	[368]
		Development of MS21, a VHL-recruiting, pan-pAKT targeting PROTAC derived from the ATP-competitive inhibitor, AZD5363, which reduced both cell and tumor growth in mutant PI3K-PTEN and wild-type, but not mutant, KRAS/BRAF cell lines, by destabilizing AURKB and arresting cells in the G2-M phase.	[408]
		Development of MS15, a pan-AKT, allosteric inhibitor-based PROTAC, which potently and selectively degraded AKT, and inhibited the growth of both PI3K-PTEN and KRAS/BRAF-mutant cancer cells.	[409]
		The discovery of additional pan-AKT targeting, VHL-and CRBN- recruiting PROTACs, MS143, MS98, MS5033, and MS170 using SAR, which inhibited AKT downstream signaling and cancer cell proliferation. MS143, in particular, showed superior anti-growth properties compared with AZD5363. All four drugs additionally demonstrated adequate plasma exposure levels in mice. MS143 was also effective in suppressing tumor growth in mice without causing any appreciable toxicity.	[410, 411]
		Using an in silico modeling approach to design a unique pan-AKT, CRBN-recruiting PROTAC, B4, that has a pyrazole-furan conjugated piperidine derived AKT-targeting moiety. B4 potently inhibited AKT downstream signaling and demonstrated efficacy against hematological cancers.	[412]
		Development of WJ112-14, a CRBN-recruiting, pan-class I PI3K isoform binding module that reduced off-target effects by selectively degrading PI3Kα in cancer cells.	[413]
Nanobodies	Specific binding to dysregulated or over-expressed oncogenic proteins in tumor cells to block their activity or trigger their degradation.	Development of AKT1- and AKT2-specific nanobodies to dissect their isoform-specific functions, and inhibit their interaction with PIP3.	[389, 414]
		Development of the hydrophobic motif-targeting AKT2 nanobodies, Nb8 and Nb9, which decreased MDA-MB-231 cell growth and viability by decreasing AKT activation and expression/phosphorylation of downstream AKT targets, decreasing the number of focal adhesions and stress fibers, and inducing cell cycle arrest and autophagy.	[390]

*Legend*: *POI* protein of interest, *CRBN* cereblon, *AURKB* aurora kinase B,* PROTAC* proteolysis-targeting chimera, *PI3K* phosphatidylinositol-3 kinase, *PIP3* phosphatidylinositol (3, 4, 5)-trisphosphate, *PTEN* phosphatase and tensin homolog, *KRAS* Kirsten rat sarcoma viral oncogene homolog, *VHL* Von Hippel-Lindau, *BRAF* v-raf murine sarcoma viral oncogene homolog B1, *SAR* structure-activity relationship

Nanoparticles can act as a delivery vehicle not only for nucleic acids, but for drugs and proteins as well. Distinctive features of nanoparticle-based delivery systems that make them a burgeoning platform for cancer treatment is their biocompatibility, stability in the circulation, enhanced permeability and retention effect in tissues, specific cellular targeting, membrane traversal, intracellular target localization, sustained drug release and superior cytotoxic capabilities [415–417]. Another advantage is that nanoparticles themselves can be conjugated to aptamers for targeted delivery; Gonzalez-Valdivieso and colleagues devised a docetaxel and AKT peptide inhibitor recombinant fusion-containing elastin-like recombinamer (ELR) vehicle, which was conjugated to a DNA aptamer that specifically recognizes the tumor marker, CD44, to selectively target colorectal cancer cells [418]. Tumor selectivity has also been endeavored through the use of pH-sensitive smart cancer nano-theranostics that home to the acidic tumor microenvironment (TME) [419]. The simultaneous delivery of multiple drugs is another advantage of nanotherapeutics, with antibody-conjugated drug-loaded nanotherapeutics (ADN) being a significant advancement in the field of immunotherapy [420]. The approach of using an anti-CD47 and anti-PDL1 antibody pair conjugated to the surface of a nanoparticle encasing a PI3K-AKT-mTOR inhibitor, proved to be more efficacious in reducing tumor burden in a non-small cell lung cancer immunocompetent mouse model, compared with current approaches using a PDL1 inhibitor [420]. Perhaps designing aptamer-conjugated, or microenvironment- or stimuli-sensitive ADN, for targeted delivery, can overcome the limitations of RNA- and protein-based degraders for AKT isoform-targeting, especially given the well-established role of AKT in immune evasion [9] and the effect of different AKT isoforms on immune cells, as discussed above.

Designing isoform-specific drugs having mutant or allele selectivity, such as inhibitors that target AKT1 E17K (https://www.rcsb.org/structure/8uw9), can potentially result in an even greater reduction in off-target effects, similar to drugs targeting the mutant form of PI3Kα, which delayed the onset of rash and hyperglycemia in patient-derived tumor xenograft models [421].

An indirect, novel approach to targeting AKT would be to target proteotoxic stress imparted by AKT hyperactivation due to ongoing protein synthesis. Typically, 30% or more of newly synthesized proteins in cells are immediately recycled due to folding or translation errors [422]: this percentage increases depending on various extrinsic and intrinsic cellular cues [423]. Under proteotoxic stress conditions, cells deploy defense mechanisms to help mitigate this stress and restore homeostasis. If stress-mitigating factors are absent or the cell exceeds its threshold of stress tolerability, cell death ensues [424, 425]. When cells are subjected to hyperthermia, for example, protein unfolding occurs. This activates the transcription factor HSF-1, which upregulates the expression of chaperone proteins that recycle unfolded proteins or assist them with refolding [426]. Moreover, the accumulation of unfolded proteins in the endoplasmic reticulum results in ‘ER stress’, which causes the cell to halt protein synthesis and unleash an unfolded protein response through PERK, IRE1α, and ATF6 [422, 427]. ATF6 induces the transcription of the *XBP1* gene [428], while IRE1α orchestrates the unconventional splicing of *ATF6* mRNA, creating an open reading frame (ORF) that is translated into a shorter version of XBP1, known as XBP1s [429]. XBP1s functions as a transcription factor that, like HSF-1, induces the expression of chaperone proteins that help combat ER stress [430]. Cells harboring hyperactive AKT or loss of PTEN displayed elevated levels of XBP1 and HSF-1, were more sensitive to heat shock, and depended on XBP1 for growth, suggesting that XBP1 is a therapeutic vulnerability in AKT-hyperactivated tumors [423].

Increased glycolytic shuttling of glucose and mitochondrial metabolism are other novel targetable features of cells with hyperactivated AKT. In mouse models of PTEN-deficient prostate cancer, combining rapamycin with a ROS inducer causes tumor regression, prolongs survival, and sensitizes tumor cells to ROS-induced cell death by tilting the balance towards redox stress and overwhelming ROS scavengers. A similar result is observed when the hexokinase-2 gene (*HK2*) is deleted [431]. Translating these findings to the clinic may only sometimes be feasible, however.

Autophagy is an area of intense research in the field of cancer biology. It is a double-edged sword in that it can promote or suppress tumorigenesis, depending on the cellular context; blocking autophagy at a late stage has been shown to induce cell death, according to multiple studies [432–436]. Autophagy is activated by nutrient deprivation, the accumulation of abnormal proteins, or organelle damage, and involves the formation of autophagosomes that encircle the components to be degraded; autophagosomes then fuse with lysosomes, forming autophagolysosomes, which are digested and recycled [436]. Combining an AKT inhibitor with a lysosomotropic agent in AKT-hyperactivated cells to block autophagy is another therapeutic strategy that may warrant further investigation. AKT inhibition alone is enough to activate autophagy, either by increasing ER stress, increasing ROS formation and mitochondrial damage (mitophagy), activating FOXO proteins, decreasing glucose and mitochondrial metabolism, or inhibiting the mTORC1 complex, and that in itself can cause cell death, either via apoptosis or self-digestion. However, PC3 cells expressing shRNA against Akt1/2/3 can survive, even under serum-starved conditions, and when grown as xenograft tumors, can develop after a period of tumor regression, suggesting that autophagy induced by Akt inhibition protects against cell death. This was corroborated when it was observed that treating cells with an AKT inhibitor and chloroquine, a lysosomotropic agent that blocks autolysosomal digestion, resulted in an increase in apoptotic nuclei, caspase-3 activation and an increase in the size of autophagic vesicles.

Interestingly, the authors observed an increase in mitochondrial superoxide and cellular ROS levels upon treatment with AKT inhibitors alone, which was resolved shortly after that, whereas co-treatment with chloroquine resulted in sustained ROS generation. Treating cells with a ROS scavenger inhibited autophagy caused by AKT inhibition and prevented cell death, leading the investigators to conclude that under autophagy-inducing conditions caused by AKT inhibition, PC3 cells employ autophagy as a pro-survival mechanism to prevent the aggregation of ROS generators that can accentuate ROS damage, causing both apoptotic and non-apoptotic cell death [437, 438]. However, a phase I trial to assess the tolerability and safety of MK2206 with hydroxychloroquine for the treatment of advanced solid tumors reported minimal anti-tumoral activity with many drug-related adverse effects [439].

Chromatin modifiers, such as lysine methyltransferase inhibitors and histone deacetylase inhibitors, may prevent the activation of AKT via post-translational modification. They may also have the benefit of increasing or altering the expression of tumor suppressive genes, including those that negatively regulate AKT. AKT normally forms a complex with the chaperone protein, HSP90, which is required for its structural maturation and stability [440]. Utilizing HSP90 inhibitors that occupy the ATP-binding pockets of these proteins can shorten the half-life of AKT and decrease its expression, which is noteworthy from a therapeutics perspective (reviewed in [441]).

## Biomarkers predicting sensitivity and response to AKT inhibitors

Previously, numerous clinical trials were undertaken to try to identify biomarkers of sensitivity and response to AKT inhibitors, but the outcomes have been mixed, and there appears to be a discord between alterations in the PI3K-AKT pathway and response to AKT inhibitors. In a nonrandomized trial of patients with AKT1 E17K-mutated metastatic histologically variable tumors, for example, treatment with capivasertib only mildly affected pS6 and PTEN phosphorylation [442], while in the STAKT trial, a two-stage, double-blind, randomized, placebo-controlled study conducted in patients with ER + breast cancer, a decrease in the level of pGSK3β, pPRAS40, pS6, a paradoxical increase in pAKT, and an increase in nuclear FOXO3A from baseline (the latter two findings being consistent with the mechanism of capivasertib) were observed in the capivasertib-treated group versus the placebo group (n = 11) [305]. In phase II, randomized, multicenter, I-SPY2 trial, in which patients with early hormone receptor (HR)-negative/HER-2 positive breast cancer and triple-negative breast cancer (TNBC) received neoadjuvant treatment with MK-2206 plus standard therapy (versus placebo plus standard therapy), pathological complete responses were associated with high pre-treatment levels of pAKT, pSGK, pmTOR, and pTSC2, in the HER-2 positive subset only, as determined by phosphoproteomic analyses. In the TNBC group, however, patients with more significant pathological responses had lower levels of the corresponding biomarkers (pAKT, pmTOR, and pTSC2) [443]. In two randomized, phase II trials, LOTUS and PAKT, an increase in progression-free survival (PFS) was observed in TNBC patients with PIK3CA/AKT/PTEN alterations who were treated with either capivasertib or ipatasertib and paclitaxel, but this improvement in PFS was not observed in PTEN-low patients in the LOTUS cohort [310, 312] or in a phase III randomized trial that tested the same combination of therapies in a similar group of TNBC patients [444].

Until recently, the only reliable predictive biomarker of sensitivity to most ATP-competitive inhibitors but not allosteric inhibitors, was the *AKT1* E17K missense mutation, based on the results of a multi-histology basket study of capivasertib in patients with advanced gynecologic, ER-positive breast cancer, and other solid tumors [445]. The number of AKT mutant alleles displaying sensitivity to ATP-competitive inhibitors (capivasertib) has now been expanded to include a slew of non-E17K (missense) activating *AKT1/2* mutations, resulting from small in-frame duplications (indels) that induce structural conformations in AKT different from activating missense mutations and activate the PI3K-AKT pathway to a much greater degree. Cells with AKT in-del mutations showed heightened sensitivity to ATP-competitive inhibitors compared to activating missense mutations, which showed a varied response to allosteric and ATP-competitive inhibitors. Interestingly, cells expressing *AKT1* and *AKT2* in-del mutants were resistant to allosteric inhibitors, likely due to the structural displacement of this drug class at the PH-kinase interface induced by the in-del mutation. In an agnostic clinical trial initiated by the same investigators, it was found that patients with different tumor lineages harboring rare activating *AKT1-3* mutant variants, including but not limited to activating in-dels, responded to capivasertib, broadening the list of biomarkers that predict sensitivity to ATP-competitive inhibitors [446].

The real challenge lies in identifying isoform-specific substrates that can be utilized not only as targetable biomarkers but also to predict isoform treatment sensitivity and gauge treatment responses. This would also enable the understanding of how targeting the PI3K-AKT pathway affects upstream receptor and non-receptor tyrosine kinases, PI3K isoform, and AKT isoform activity in the case of therapeutic resistance, for example. However, this is no easy feat.

A successful example of biomarker-driven AKT therapeutics is the recent breakthrough by Craven et al. who showed that the mutant lysine in AKT1 E17K can be targeted by a covalent allosteric salicylaldehyde-based inhibitor that recruits endogenous Zn2+; by sparing collateral AKT2 inhibition, it is anticipated that this isoform-specific and mutant-selective inhibitor will result in decreased side effects, including hyperglycemia, in patients [448].

## Conclusions and future perspectives

It is the authors’ opinion that the war on hyperactivated AKT in cancer will be best waged in the future using a combination of AKT degraders, preferably those with isoform-selectivity, in a cancer-type and context-specific manner, and immunotherapy, in the form of CAR-T cell therapy (with ex vivo manipulation of CAR-T cells by targeting specific isoforms), or immune checkpoint inhibitors, which, in theory, can subdue tumor cells and boost tumoral immunogenicity by overriding the immunosuppressive TME. However, we still have many hurdles to cross before this can be made a reality. In addition, the advancement of drugs, such as capivasertib, to phase III trials means that AKT inhibitors, despite their shortcomings, have the potential to have a positive impact on breast cancer and potentially other cancer types and offer a glimmer of hope to patients living with the disease, who will now be able to reap the benefits of this drug. Just recently (November 16, 2023), the FDA approved capivasertib (Truqap, AstraZeneca Pharmaceuticals) with fulvestrant for adult patients with HR-positive, HER2-negative locally advanced or metastatic breast cancer with one or more *PIK3CA/AKT1/PTEN* alterations, as detected by an FDA-approved test, following progression on at least one endocrine-based regimen in the metastatic setting or recurrence on or within 12 months of completing adjuvant therapy. In addition, we believe that the recent development of a mutant-specific allosteric inhibitor will be a game-changer in the field of AKT therapeutics. Thus, despite the many challenges, the future of AKT inhibitors in the oncology clinic is bright.

## Abbreviations

SRC 2: Src homology 2
PKB: Protein kinase B
AGC: Protein kinases A, G, and C
SGK: Serum-glucocorticoid regulated kinase
PH: Pleckstrin homology
Glut4: Glucose transporter type 4
TCL: T-cell leukemia-1
TCL1b: T-cell leukemia-1b
PI3K: Phosphatidylinositol-3-phosphate kinase
PIP2: Phosphatidylinositol-4,5-bisphosphate
PIP3: Phosphatidylinositol-3,4,5-triphosphate
TRAF6: Tumor necrosis factor receptor associated factor 6
TRAF4: Tumor necrosis factor receptor associated factor 4
JIP1: JNK-interacting protein 1
SKP2: S-phase kinase associated protein 2
SETDB-1: SET domain bifurcated histone lysine methyltransferase 1
CYLD: CYLD lysine 63 deubiquitinase
PDK1: 3-Phosphoinositide-dependent protein kinase 1
PDK2: Phosphoinositide-dependent protein kinase 2
mTORC1: Mammalian target of rapamycin complex 1
mTORC2: Mammalian target of rapamycin complex 2
InsR: Insulin receptor
IGF1R: Insulin-like growth factor receptor
PRK-2: Protein kinase c-related kinase 2
DNA-PK: DNA-dependent protein kinase
ATM: Ataxia telangiectasia mutated
GEF: Guanine exchange factor
ECT2: Epithelial sequence transforming sequence 2
IKKE: I B kinase epsilon
TBK1: TANK-binding kinase 1
CTMP: Carboxyl terminal modulatory protein
MMTV: Mouse mammary tumor virus
CDK2: Cyclin-dependent kinase 2
Erk 1/2: Extracellular signal-regulated protein kinase ½
Hsp 90: Heat shock protein 90
Hsp 27: Heat shock protein 27
TSC1/2: Tuberous sclerosis 1 and 2
Rheb: Ras homolog enriched in the brain
4E-BP1: Eukaryotic translation initiation factor 4E (eIF4E) binding protein-1
IKK: I B kinase
Mdm2: Mouse double minute 2 homolog
Bad: BCL-2 associated agonist of cell death (Bad)
GSK3: Glycogen synthase kinase-3
FOXO: Forkhead box O
TRF1: Telomeric repeat binding factor 1
PP2A: Protein phosphatase 2A
PHLPP1/2: PH domain leucine-rich repeat-containing protein phosphatase ½
HM: Hydrophobic motif
A-loop: Activation loop
PTEN: Phosphatase and tensin homolog
SHIP: Src homology 2 domain-containing inositol-5-phosphatase
FKBP51: FK506 binding protein 51
RACK1: Receptor for protein kinase 1
MASTL: Microtubule-associated serine/threonine kinase-like
I1PP2A: Inhibitor 1 of PP2A
I2PP2A: Inhibitor 2 of PP2A
CIP2A: Cellular inhibitor of PP2A
SENP: Sentrin-specific protease
HSPA5: Heat shock protein family A member 5
USP7: Ubiquitin-specific peptidase 7
BAP1: BRCA1-associated protein 1
ASXL1: Additional sex combs-like protein 1
IRS1/2: Insulin receptor substrate ½
MAPK: Mitogen activated protein kinase
TKI: Tyrosine kinase inhibitor
RTK: Receptor tyrosine kinase
KRAS: Kirsten rat sarcoma virus
RAS: Rat sarcoma
ROS: Reactive oxygen species
EGFR: Epidermal growth factor receptor
NF-κB: Nuclear factor kappa B
Wnt: Wingless/integrated
PLD1: Phospholipase D1
ASK1: Apoptosis signal-regulated kinase 1
JNK: C-Jun N-terminal kinase
ATR: Ataxia telangiectasia and Rad3-related
Chk1: Checkpoint kinase 1
RUNX2: Runt-related transcription factor 2
Sp1: Specificity protein 1
GDNF: Glial cell-line derived neurotrophic factor
ST3GAL1: ST3 Beta-Galactoside Alpha-2,3-Sialyltransferase 1
NF1: Neurofibromin 1
OPA: Ovine pulmonary adenocarcinoma
JSRV: Jaagsiekte sheep retrovirus
SPA: Surfactant protein A
SFB: Surfactant protein B
LTR: Long terminal repeats
HNF-3β: Hepatocyte nuclear factor 3 beta
UTR: Untranslated regions
MAGI-2: MAGUK Inverted 2
CAMK2N1: Calcium/calmodulin dependent protein kinase II inhibitor 1
LncRNA: Long non-coding RNA
HK-1: Hexokinase-1
ecRNA: Endogenous competing RNA
MERTK: Mer tyrosine kinase
Ubc9: Ubiquitin conjugating enzyme 9
SAE1: SUMO-activating enzyme subunit 1
PIAS1: SUMO ligase protein inhibitor of activated STAT1
SIRT7: Sirtuin 7
SIRT1: Sirtuin 1
HIF: Hypoxia inducible factor
EglN1: Egl-9 family hypoxia-inducible factor 1
VHL: Von Hippel-Lindau
cAMP: Cyclic adenosine monophosphate
EPAC: Exchange protein directly activated by cAMP
SFKs: Src-family kinases
PyMT: Polyomavirus middle T-ag
shRNA: Short hairpin RNA
miRNA: MicroRNA
ER: Estrogen receptor
TCR: T-cell receptor
HnRNP: Heterogenous nuclear ribonucleoprotein
PD-1: Programmed cell death protein 1
PDL-1: Programmed death ligand 1
TIL: Tumor infiltrating lymphocytes
NSG: NOD Scid gamma
CTL: Cytotoxic T lymphocytes
CAR-T: Chimeric antigen receptor T-cell
Treg: Regulatory T cells
iTreg: Induced regulatory T cells
EAE: Experimental autoimmune encephalomyelitis
Th1: T helper type 1 cells
Th2: T helper type 2 cells
TME: Tumor microenvironment
MS: Multiple sclerosis
TGF: Transforming growth factor beta
M1 macrophages: Classically activated macrophages
M2 macrophages: Alternatively activated macrophages
ICAM-1: Intercellular adhesion molecule-1
siRNA: Small interfering RNA
CSF-1: Colony stimulating factor 1
LIMK: LIM domain kinase
DSS: Dextran sodium sulfate
IBD: Inflammatory bowel disease
HSC: Hepatic stellate cell
ALD: Alcoholic liver disease
LPS: Liposaccharide
CAAI: Covalent allosteric Akt inhibitor
HCC: Hepatocellular carcinoma
Mcl-1: Myeloid cell leukemia 1
BRCA1: Breast cancer gene 1
MERIT40: Mediator of Rap80 Interactions and Targeting 40 kDa
HER2: Human epidermal growth factor receptor 2
PR: Partial response
GC: Gastric cancer
VICTORY: Targeted agent eValuation In gastric cancer basket KORea trial
TNBC: Triple negative breast cancer
In-dels: Insertions-deletions
PROTACS: Proteolysis targeting chimeras
RIBOTACs: Ribonuclease targeting chimeras
PINAD: Proximity-induced nucleic acid degraders
PIM2: Proviral Integration site for Moloney murine leukemia virus 2
RSK: Ribosomal S6 kinase
ACLY: ATP citrate lyase
BCKDK: Branched-Chain Ketoacid Dehydrogenase Kinase
P21WAF1: P21, Wild-type p53-Activated Fragment 1
LC–MS/MS: Liquid chromatography tandem mass spectrometry
IWS1: Interacts with Spt6
HSF-1: Heat shock factor 1
PERK: Protein kinase R-like Endoplasmic Reticulum Kinase
IRE1α: Inositol-Requiring Enzyme 1 alpha
ATF6: Activating transcription factor 6
XBP1: X-box Binding Protein 1
HK-2: Hexokinase 2
FDA: Food & drug administration
